# Adaptive Flexible Sialylated Nanogels as Highly Potent Influenza A Virus Inhibitors

**DOI:** 10.1002/anie.202006145

**Published:** 2020-06-30

**Authors:** Sumati Bhatia, Malte Hilsch, Jose Luis Cuellar‐Camacho, Kai Ludwig, Chuanxiong Nie, Badri Parshad, Matthias Wallert, Stephan Block, Daniel Lauster, Christoph Böttcher, Andreas Herrmann, Rainer Haag

**Affiliations:** ^1^ Institute of Chemistry and Biochemistry Freie Universität Berlin Takustraße 3 14195 Berlin Germany; ^2^ Institute of Biology & IRI Life Sciences Humboldt-Universität zu Berlin Invalidenstraße 42 10115 Berlin Germany; ^3^ Forschungszentrum für Elektronenmikroskopie, and Core Facility BioSupraMol Institute of Chemistry and Biochemistry Freie Universität Berlin Fabeckstr. 36a 14195 Berlin Germany

**Keywords:** flexibility, infection inhibition, influenza A virus, polyglycerols, sialylated nanogels

## Abstract

Flexible multivalent 3D nanosystems that can deform and adapt onto the virus surface via specific ligand–receptor multivalent interactions can efficiently block virus adhesion onto the cell. We here report on the synthesis of a 250 nm sized flexible sialylated nanogel that adapts onto the influenza A virus (IAV) surface via multivalent binding of its sialic acid (SA) residues with hemagglutinin spike proteins on the virus surface. We could demonstrate that the high flexibility of sialylated nanogel improves IAV inhibition by 400 times as compared to a rigid sialylated nanogel in the hemagglutination inhibition assay. The flexible sialylated nanogel efficiently inhibits the influenza A/X31 (H3N2) infection with IC_50_ values in low picomolar concentrations and also blocks the virus entry into MDCK‐II cells.

## Graphical abstract

**Fighting influenza**: Through the combination of flexibility and multivalent binding, a sialylated nanogel is able to bind and adapt onto the influenza A virus surface. The resulting flexible nanogel efficiently blocks the virus binding onto the cell and inhibits infection at low pm concentrations.

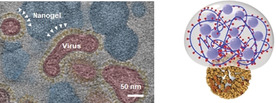

While the SARS‐CoV‐2 pandemic has impacted public health globally,^1^, ^2^ developing new concepts against respiratory viruses is of great interest. Influenza A virus (IAV) infection has been a leading cause of severe illness and mortality worldwide.^3^ IAV enters the cell by multivalent binding of its trimeric spike hemagglutinin (HA) proteins to the exposed sialic acid (SA) residues of the glycocalyx on the host cell surface.^4^ The monovalent binding affinity of HA for SA is low (≈2–4 mm), but multivalent interactions lead to a strong adhesion of virus particles on the cell surface.^5^, ^6^ IAVs are pleomorphic depending on the strain type and infection situations.^7^ They appear in a size range between 70–120 nm when spherical and up to several micrometers in length when filamentous.^8^, ^9^, ^10^ Therefore our hypothesis is that medium‐sized flexible multivalent nanoparticles of 250 nm, which can adapt to the virus surface with a large contact area and multivalent binding, could be efficient for blocking IAV particles. Such flexible sialylated nanoparticles will not only bind but also sterically shield the virus, thereby reducing virus–cell adhesion.

Various flexible nanosized functionalized scaffolds have been employed for pathogen inhibition such as, for example, graphene sheets wrapping *E. coli* bacteria.^11^ Also graphene sheets^12^ and sulfated nanogel particles^13^ inhibited the entry of Herpes simplex virus into host cells, showing the potential of flexible inhibitors that are equal in size to or larger than the pathogen.

To ensure efficient IAV inhibition by medium‐sized flexible 3D multivalent structures, an optimum SA density and accessibility to the viral surface proteins are the foremost conditions to be considered. Different carrier scaffolds including polymers,^14^, ^15^, ^16^, ^17^ small nanoparticles,^18^, ^19^ and proteins^20^ bearing multivalent SA residues have been used to inhibit IAV. Our previous report based on biocompatible polyglycerol sialosides (PGSA) has revealed that the SA density of 15–20 % for dendritic and 40–70 % for linear PGSAs is optimum for efficient IAV inhibition. The latter inhibited IAV at nanomolar concentrations and was shown to be superior to an optimized dendritic PGSA for infection inhibition both in vitro and in vivo.^21^

Here we present a new approach for the synthesis of medium‐sized flexible multivalent nanogels based on dendritic and linear polyglycerol sialosides (dPGSAN_3_ and LPGSAN_3_) with 15 and 40 % SA residues, respectively (see the Supporting Information), which could adapt onto the virus surface and efficiently inhibit binding of influenza A/X31 (H3N2) and thus, infection of the host cells. To generate nanogels with different flexibilities, 10 kDa dPG and LPG polymers bearing 7–10 cyclooctyne groups, that is, dPG‐cyclooctyne and LPG‐cyclooctyne, were prepared as cross‐linking macromonomers (Figure 1 and the Supporting Information). The bioorthogonal Cu‐free strain‐promoted click chemistry approach^22^, ^23^ was applied in an inverse nanoprecipitation experimental setup using acetone as a co‐solvent. This surfactant‐free technique makes it possible to prepare nanoparticles in different size ranges by varying the ratio of good solvent to nonsolvent without any tedious purification steps.^24^, ^25^ Composition parameters were optimized to obtain nanoparticles of reproducible size with similar ratios of sialylated macromonomers for all nanogels (NGs) (Table 1). The combination with linear polymers was expected to result in higher flexibilities compared to the purely dendritic macromonomer based approach, but provides different accessibility of SA functional groups. Three nanogels, R‐NG 1, F‐NG 2, and F‐NG 3 were prepared in quantitative yields by cross‐linking of (dPGSAN_3_) with (dPG‐cyclooctyne), (dPGSAN_3_) with (LPG‐cyclooctyne), and (LPGSAN_3_) with (dPG‐cyclooctyne). All three nanogels were purified by dialysis in H_2_O and characterized by ^1^H NMR spectroscopy (Figures S9–S11). According to elemental analysis, all nanogels had similar sialylated dPGSA or LPGSA polymer content by weight (≈65 %). A negative control NG (C‐NG) without any SA and with a hydrodynamic diameter (D_H_) of 230 nm was prepared by cross‐linking of dPG(N_3_)_10 %_ and dPG‐(cyclooctyne)_10 %_ (see the Supporting Information).

**Figure 1:**
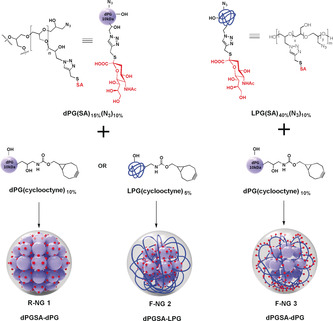
Preparation of different nanogels by inverse nanoprecipitation in acetone. Aqueous solutions of sialylated polymers bearing azide groups and respective polymers bearing multiple cyclooctyne groups are mixed and cross‐linked in situ.

**Table 1:** Composition, size, and zeta‐potential of different nanogels.

Nanogel	Macromonomers				H_2_O	CH_3_COCH_3_	Size by DLS^[a]^ (d±SD) [nm]	PDI^[b]^	Size by NTA (d±SD) [nm]	Zeta‐ potential^[a]^ [mV]	PGSA content^[c]^ [wt %]
	dPGSA 20 wt % solution	LPGSA 10 wt % solution	dPG‐cyclooctyne 10 wt % solution	LPG‐cyclooctyne 10 wt % solution							
R‐NG 1	50 μL	–	150 μL	–	5 mL	200 mL	283.0±1.3	0.13	260.1±82.8	−12.0±0.5	62.7
F‐NG 2	50 μL	–	–	150 μL	5 mL	200 mL	250.9±0.8	0.13	226.9±86.3	−7.8±0.8	68.7
F‐NG 3	–	50 μL	75 μL	–	2 mL	100 mL	256.5±5.6	0.15	230.8±82.5	−18.9±0.9	61.1

[a] In PBS (pH 7.4, 10 mm) at 1 mg mL^−1^. [b] Polydispersity index obtained by DLS. [c] Determined by elemental analysis of lyophilized nanogels.

All three nanogels showed unimodal size distribution with a low polydipersity index and a D_H_ between 250–283 nm as observed by dynamic light scattering (DLS) (Figure 2 a, Figures S12 and S13) and in agreement with nanoparticle tracking analysis (NTA) (Table 1). NTA analysis also provided insight into the number of particles per weight. For each nanogel, 1 μg mL^−1^ is equal to about 10^8^ particles mL^−1^ (Figure S26, Table S1). This roughly corresponds to a molecular weight in the GDa (10^9^ Da) range for all NGs. Negative zeta potential (ξ) values of functionalized gels were related to the SA exposition on the surface of NGs. Both R‐NG 1 and F‐NG 2 had similar content of dPGSA macromonomers. The fact that the ξ value of F‐NG 2 (−7.8 mV) is lower than that of R‐NG 1 (−12.0 mV) indicated that most of the SA residues are hidden by LPG coils after cross‐linking with LPG‐cyclooct macromonomer. The F‐NG 3 prepared by LPGSA macromonomer exhibited the highest ξ of −18.0 mV. This indicates a high exposure of SA residues on the F‐NG 3 surface (Table 1).

**Figure 2:**
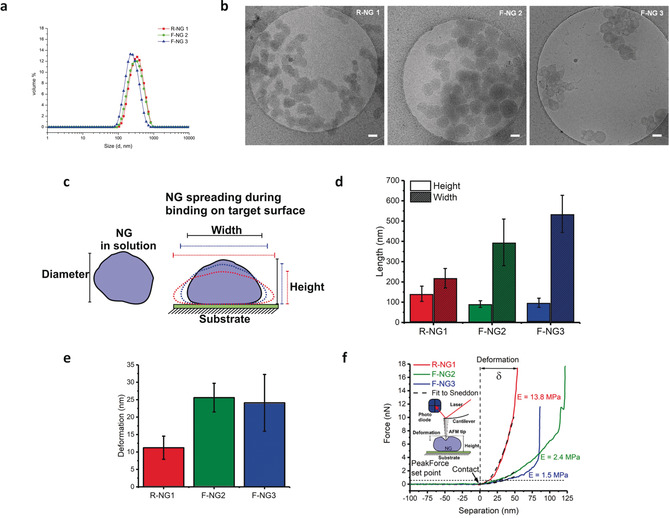
a) Volume distribution profiles by DLS for three nanogels in PBS (pH 7.4, 10 mm) at 1 mg mL^−1^. b) Morphology of nanogels embedded in vitreous ice: cryo‐TEM images of R‐NG 1, F‐NG 2, and F‐NG 3 in PBS pH 7.4. Scale bar: 100 nm. c) Schematic representation of a NG in solution and after binding to a substrate as required for AFM measurements. d) Height and width measured from profiles of single NGs. F‐NG 2 and F‐NG 3 showed lower heights and larger widths than R‐NG 1. e) NG deformation quantified by PeakForce mode. f) Three representative force–separation curves obtained by point nanoindentation on individual NGs with a fit using the Sneddon model to obtain the NG Young's modulus. Inset shows a depiction of the indentation process by AFM and the horizontal dashed line shows the maximum PeakForce set point used during imaging.

The mechanical properties of NGs were studied by stable sample immobilization on a substrate by atomic force microscopy (AFM) in PeakForce QNM (Quantitative NanoMechanics) mode (Figure 2 c). The technique has been applied to quantify the elasticity and deformation capacity of soft NGs and nanocapsules in solution.^13^, ^26^, ^27^ In PeakForce mode, the AFM tip repeatedly pushes the sample surface with a constant maximal force at each point being scanned. Consequently, a difference in deformation depth by the tip is expected for NGs with different material properties (Figure S22). The PeakForce mode is used to determine the deformability of soft materials.

Height and deformation maps for all three NGs R‐NG 1, F‐NG 2, and F‐NG 3 immobilized on a positively charged surface were obtained (Figures S23–S25). Cross section profiles for height and deformation indicate only minor differences in surface morphology and the structure of NGs, but an evident difference in deformation between R‐NG 1 and F‐NG 2 as well as F‐NG 3, indicating that the tip penetrates less into the rigid R‐NG 1. For soft nanoparticles with a quasi‐spherical shape in solution, binding or adsorption onto a target surface inherently induces a certain degree of flattening of the particle (reduction in height, h) together with a spreading increasing the contact area (increase in width, w) (Figure 2 c). The higher flexibility of F‐NG 2 and F‐NG 3 as compared to the more rigid R‐NG 1 was supported by their larger spreading capacity on the substrate (Figure 2 d). Tip penetration/deformation (Figure 2 e) confirms that F‐NG 2 and F‐NG 3 are indeed more prone to deformation than R‐NG 1. Estimation of the NG Young's modulus (E) can be made by the Sneddon model for the compression of the sample surface by a conical indentor (see the Supporting Information). We obtained 12.7±8.8 MPa for R‐NG 1, in contrast to 2.4±0.8 MPa and 3.1±2.3 MPa for F‐NG 2 and F‐NG 3, respectively. Single point nanoindentations were applied to verify the extent of deformation obtained by PeakForce nanomechanical mapping and to observe the path of applied force as a function of the tip–NG separation (Figure 2 f). Fitting the data for the indentation of individual NGs with the Sneddon model reveals values within the range of those obtained from nanomechanical mapping. Comparing our results for NG spreading, both PeakForce nanomechanical mapping and point nanoindentations suggest that our cross‐linking approach renders F‐NG 2 and F‐NG 3 more flexible than R‐NG 1.

In order to prove the IAV interaction, cryo‐TEM and cryo‐electron tomography (cryo‐ET) were used to visualize the binding of the seasonal influenza A/X31 (H3N2) to NGs. All three NGs have an almost globular shape and are in the range of 100 to 200 nm in size (Figures 2 b). They interact with virus surfaces, but to different degrees. For R‐NG 1 and even more pronounced for F‐NG 3, viral particles could be seen in the immediate vicinity of NGs (Figures S15‐S17), which for the latter case was also confirmed by cryo‐ET (Figure 3 a and Figure S20). These images show a certain degree of deformation and adaptation of the flexible NG particles while binding onto the IAV surface. Such binding events for F‐NG 2 could be observed much less frequently (Figure S16). The control NG (C‐NG) without SA residues did not show any binding event with virus particles (Figure S19). The cryo‐TEM analysis thus provided a quasi‐snapshot of R‐NG 1 and F‐NG 3 virus binding, which was confirmed by the biological tests.

**Figure 3:**
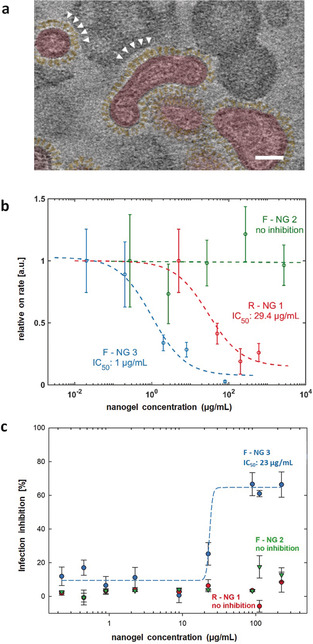
a) A cross‐section (0.75 nm thick slice) through the reconstructed 3D volume of a cryo‐electron tomograph of F‐NG 3 incubated with influenza A/X31 in PBS pH 7.4 for 30 min at RT (see also Figure S20). The viral particles are colored in red for identification and the spike proteins are in yellow, accordingly. White arrowheads mark the contact zone of the viral spikes with the flexible nanogels. The scale bar corresponds to 50 nm. b) On‐rate binding plots of influenza A/X31 with GD1a (1 mol %) receptors within a supported lipid bilayer in the presence of R‐NG 1, F‐NG 2, and F‐NG 3 as measured by TIRF microscopy. c) Inhibition of infection of MDCK‐II cells by influenza A/X31 (MOI 0.5) pretreated with R‐NG 1, F‐NG 2, and F‐NG 3. Inhibition of infection was measured 48 h p.i. Virus‐induced cytopathicity was assessed by a CellTiter Aqueous One Solution (Promega) based viability assay. IC_50_ values were obtained by fitting plots by a four‐parametric sigmoidal function. Error bars: SEM (*n*≥3).

Next, the potential of these multivalent sialylated NGs to prevent binding of the seasonal influenza A/X31 to cells was studied by hemagglutination inhibition (HAI). Concentrations of R‐NG 1 and F‐NG 3 showing binding inhibition were 200 and 0.5 μg mL^−1^, respectively. Based on the molecular weight approximations using NTA, this corresponds to about 200 pm and 500 fm for R‐NG 1 and F‐NG 3, respectively. F‐NG 2 and the control C‐NG did not cause any inhibition up to the maximum tested concentration of 600 μg mL^−1^. Though expected for C‐NG it is surprising for F‐NG 2. A potential explanation is that for F‐NG 2, SA residues could be shielded by the long LPG chains without SAs, as shown in Figure 1. This is also indicated by the rather low ξ value, which may explain the low binding potential of F‐NG 2.

Total internal reflection fluorescence (TIRF) microscopy was used to extract IC_50_ values of NGs for their inhibition of IAV binding to a cell‐surface‐mimicking glass slide. TIRF microscopy is a powerful tool to visualize transient binding events of single viruses at appropriate interfaces.^28^, ^29^, ^30^ The evanescent wave of TIRF penetrates only about 100 nm from the interface into the sample. Hence, only dye‐labelled viruses that bind to the interface are within this evanescent wave and fluoresce due to the confined excitation, while viruses not bound to the interface are not excited and thus invisible. Here, the influence of NGs on influenza A/X31 binding to SA of GD1a‐containing supported lipid bilayer (SLB) was studied. F‐NG 2 did not show any change in the attachment rate, confirming that it does not inhibit IAV. In contrast, F‐NG 3 and R‐NG 1 decrease the attachment rate, which is indicative for inhibited binding of IAVs to GD1a‐containing SLBs. The inhibition concentrations (IC_50_) are 1.0 μg mL^−1^ (F‐NG 3) and 29.4 μg mL^−1^ (R‐NG 1), showing that F‐NG 3 has a ≈30 times lower inhibition concentration than R‐NG 1.

The HAI test and TIRF microscopy analysis proved the binding of sialylated NGs to the virus. For analyzing the infection inhibition potency of NGs, influenza A/X31 at a multiplicity of infection (MOI) of 0.5, was pretreated with the NGs for 30 min and incubated with MDCK‐II (Madin‐Darby canine kidney) cells. Infection was assessed by a MTS cell viability assay after 48 h p.i. Only F‐NG 3 showed a protective effect in a dose‐dependent manner with an IC_50_ value of 23 μg mL^−1^, which roughly corresponds to 2.3 pm nanoparticle concentration. NGs by themselves (without viruses) did not affect cell viability up to a concentration of 250 μg mL^−1^ using MDCK‐II epithelial cells (Figure S21).

In order to prove that virus binding to MDCK‐II cells is blocked by the NGs, we studied cell binding of DiO‐labelled virus by confocal laser scanning fluorescence microscopy (CLSM). Viruses were incubated with NGs for 45 min and subsequently with MDCK‐II cells for another 2 hours. Then, unbound viruses and NG were removed by washing with PBS, and Z‐stack CLSM images were acquired to visualize the viral particles attached to cells (Figure 4 a–c). The amount of DiO‐labelled viruses decreased significantly in the presence of F‐NG 3, indicating that viral binding has been blocked. From the virus counting results in images, F‐NG 3 showed significant inhibition of the binding, with 98.2±1.8 % being blocked (see the Supporting Information). Therefore, we can confirm that F‐NG 3 can significantly block the viral binding to the MDCK‐II cells, and, thus their infection already at the entry step.

**Figure 4:**
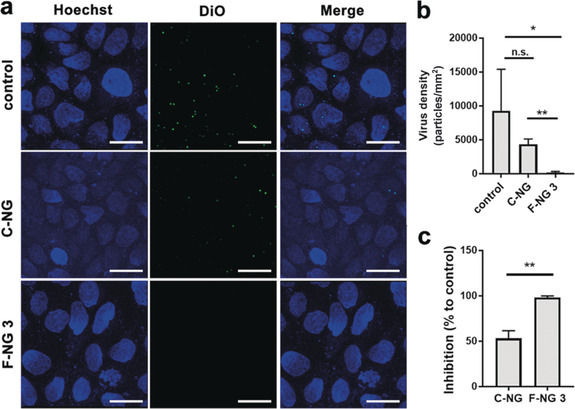
a) Z‐stacked CLSM images for the virus binding to and entry into MDCK‐II cells in the presence of the nanogel. Scale bar: 10 μm. b, c) Virus counts by pixel size from images and the corresponding inhibition ratios. Values are expressed as mean ±SD, *n*=4. n.s.: no significant difference, *p<0.05, **p<0.01 by Student t‐tests.

We could demonstrate here that the combination of deformable flexible scaffolds (F‐NGs) and multivalent presentation of influenza A virus‐specific ligands (SAs) on these scaffolds generates adaptable 3D systems that can almost completely block virus adhesion onto cells. The nanogel F‐NG 3 deforms and adapts onto the influenza A virus surface because of its high flexibility and exposed SA residues. F‐NG 3 efficiently blocks the virus adhesion on cells up to 98.2±1.8 % and also inhibits the infection at low pm concentrations in vitro. This concept might also be applied to block the entry of other respiratory viruses and eventually, reduce viral infection.

## Conflict of interest

The authors declare no conflict of interest.

## Supporting information

As a service to our authors and readers, this journal provides supporting information supplied by the authors. Such materials are peer reviewed and may be re‐organized for online delivery, but are not copy‐edited or typeset. Technical support issues arising from supporting information (other than missing files) should be addressed to the authors.

Supplementary
