# Supplementary material for: Adaptive Flexible Sialylated Nanogels as Highly Potent Influenza A Virus Inhibitors
Source: Angew Chem Int Ed Engl. 2020 Jun 30;59(30):12417–22. doi: 10.1002/anie.202006145 (PMC7384064; doi:10.1002/anie.202006145)
Supplement: Supplementary file 1 — Supplementary [file ANIE-59-12417-s001.pdf]

Angewandte Chemie
Eine Zeitschrift der Gesellschaft Deutscher Chemiker

Supporting Information

Adaptive Flexible Sialylated Nanogels as Highly Potent Influenza A Virus Inhibitors

Sumati Bhatia, Malte Hilsch, Jose Luis Cuellar-Camacho, Kai Ludwig,* Chuanxiong Nie, Badri Parshad, Matthias Wallert, Stephan Block, Daniel Lauster, Christoph Böttcher, Andreas Herrmann, and Rainer Haag**

anie_202006145_sm_miscellaneous_information.pdf

Table of contents

1. Experimental Section
1.1. Materials
1.2. Synthesis of macromonomers
1.3. Method for nanogel preparation
1.4. Cryo-TEM measurement
1.5. AFM measurement
1.6. TIRF measurement
1.7. Biological assays
1.8. Confocal studies for virus inhibition

2. Supporting figures and Tables
2.1. ^{1}H NMR of macromonomers and nanogels (Figure S1-Figure S11)
2.2. Size distribution profiles by DLS (Figure S12-S14)
2.3. Cryo-TEM of nanogels with influenza A virus (Figure S15-S20)
2.4. Cell-toxicity assay with nanogels (Figure S21)
2.5. AFM images (Figure S22-S25)
2.6. Data for confocal image analysis
2.7. Size distribution profiles by NTA (Figure S26, Table S1)

1. Experimental Section

1.1. Materials

All reagents and solvents were purchased from commercial suppliers and used without further purification. Reactions requiring dry or oxygen-free conditions were carried out under argon in Schlenk glassware. 1H spectra were recorded on Bruker AMX 500 (500 MHz) and Delta Joel Eclipse 700 (700 MHz) spectrometer at 25 ^oC and calibrated by using the deuterated solvent peak. Molecular weight distributions of LPG10, dPG10, and the post functionalized polymers were determined by means of GPC coupled to a refractive index detector (RI) for obtaining the complete distribution (Mn, Mp, Mw, dispersity). Measurements were carried out under highly diluted conditions (5 mg/ml) from a GPC consisting of an Agilent 1100 solvent delivery system with pump, manual injector, and an Agilent differential refractometer. Three 30 cm columns (PPS: Polymer Standards Service GmbH, Germany; Suprema 100 Å, 1000 Å, 3000 Å with 5 and 10 mm particle size) were used to separate aqueous polymer samples using water with 0.1 N NaNO3 as the mobile phase at a flow rate of 1 ml/min. The columns were operated at room temperature (rt) with the RI detector at 50 ^0C. The calibration was performed by using certified standards pullulan (linear) and dextran (branched) from PSS. The particle size distribution and ζ-potential of the nanogels were measured at a concentration of 1 mg/mL in phosphate-buffered saline (PBS) at 25 °C using a Zetasizer (Malvern Zetasizer-Nano ZS, Malvern Instruments Limited, Worcestershire, UK) and temperature equilibration for 60 s. The intensity, volume, and number by size distribution and the PDI values were recorded. In addition, the

hydrodynamic diameter of the nanogels was measured by NTA with a Nanosight NS500. The concentration used for NTA was 1 µg/mL. Water of Millipore quality was used in all experiments. Naturally occurring sialic acids constitute a family of more than 50 structurally distinct nine-carbon 3-deoxy-ulosonic acids, the most widespread derivative being 5-N-acetyl-neuraminic acid (Neu5Ac). We used the abbreviation of sialic acid (SA) for Neu5Ac.

1.2. Synthesis of macromonomers

$LPG_{10}OH$ (M_n = 10.1 kDa, D = 1.29) and $dPG_{10}OH$ (M_n = 10.6 kDa, D = 1.56) were used as starting polymers and prepared as reported earlier.[1,2]

Synthesis of $LPG_{10}(N_3)_{0.50}$ and $LPG_{10}(NH_2)_{0.10}$

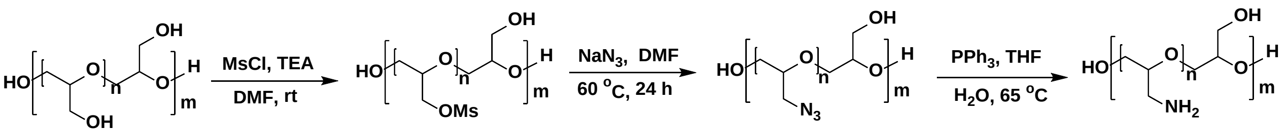

Scheme 1. Synthesis of $LPGN_3$ and $LPGNH_2$ derivatives

Synthesis of $LPG_{10}(OMs)_{0.10}$

$LPG_{10}(OMs)_{0.10}$ is synthesized by the similar procedure reported earlier by our group.[3] Dried LPG_{10}-OH (1.0 g, 13.5 mmol per monomer unit) was dissolved in dry DMF (30 mL) followed by addition of triethylamine (0.377 mL, 2.70 mmol, 1.3 equiv per OH to be functionalized) and placed in ice bath at 0 °C. Then mesyl chloride (0.136 mL, 1.75 mmol, 2.0 equiv per OH to be functionalized) was added and stirred at room temperature for 16 h. The resulting mixture was concentrated at reduced pressure and dialysed in MeOH for 2 days and then in water for 1 day to afford colourless viscous in 82% yield. ^1H NMR (500 MHz, D_2O) δ: 3.23 (s, 3H, -OMs protons), 4.53-3.64 (m, 43H, PG scaffold's CH and CH_2 protons). The degree of functionalization was obtained (DF= 0.10) by comparing the –OMs protons (3.23 ppm) with PG backbone protons (4.53-3.64 ppm).

Synthesis of $LPG_{10}(N_3)_{0.10}$

$LPG_{10}(N_3)_{0.10}$ is synthesized by the similar procedure reported earlier by our group.[3] Dried $LPG_{10}(OMs)_{0.10}$ (500 mg, 6.09 mmol per monomer unit) together with NaN_3 (198 mg, 3.05 mmol, 5.0 equiv per -OMs group) was dissolved in dry DMF (30 mL) in a 100 mL round bottom flask equipped with a reflux condenser. The reaction mixture was heated at 60 °C for 24 h. After filtering off the salts, DMF was evaporated and residue was dialysed in MeOH for 2 days and then in water for 1 day to afford the final product as colourless viscous liquid in 86% yield. ^1H NMR (500 MHz, D_2O) δ: 3.83-3.38 (m, PG scaffold's CH and CH_2 protons). IR (neat, cm^{-1}) ν: 3366, 2923, 2874, 2098, 1472, 1457, 1340, 1281, 1035.

Synthesis of $LPG_{10}(NH_2)_{0.10}$

$LPG_{10}(N_3)_{0.10}$ (100 mg, 2.59 mmol per monomer unit) was dissolved in THF:H_2O (1:1, 20 mL) and heated to 65 °C followed by addition of PPh_3 (68 mg dissolved in 1 mL of THF, 0.259 mmol, 1.0 equiv per OH to be functionalized) and stirred it for 24 h. Then added again PPh_3 (68 mg dissolved in 1 mL of THF) and repeated this for 3rd equiv of PPh_3 for 3rd day at 65 °C. The resulting mixture was concentrated at reduced pressure and precipitate formed was filtered,

washed with water several times. Evaporated the water and dialysed the sample in MeOH for 2 days and then in water for 1 day to afford $LPGNH_2$ as colourless viscous liquid in 75% yield. ^{1}H NMR (500 MHz, D_2O) δ: 3.79-3.64 (m, PG scaffold's CH and CH_2 protons).

Synthesis of $LPG_{10}(OMs)_{0.50}$

Dried LPG_{10}-OH (1.0 g, 13.5 mmol per monomer unit) was dissolved in dry DMF (30 mL) followed by addition of triethylamine (1.88 mL, 13.5 mmol, 1.3 equiv per OH to be functionalized) and placed in ice bath at 0 °C. Then mesyl chloride (0.68 mL, 8.78 mmol, 2.0 equiv per OH to be functionalized) was added and stirred at room temperature for 16 h. The resulting mixture was concentrated at reduced pressure and dialysed in MeOH for 2 days and then in water for 1 day to afford colourless viscous in 78% yield. ^{1}H NMR (500 MHz, D_2O) δ: 3.28 (m, 1.44H, -OMs protons), 4.61-3.50 (m, 5H, PG scaffold's CH and CH_2 protons). The degree of functionalization was obtained (DF= 0.50) by comparing the –OMs protons (3.23 ppm) with PG backbone protons (4.61-3.50).

Synthesis of $LPG_{10}(N_3)_{0.50}$

Dried $LPG_{10}(OMs)_{0.10}$ (500 mg, 4.42 mmol per monomer unit) and NaN_3 (718 mg, 11.05 mmol, 5.0 equiv per -OMs group) were dissolved together in dry DMF (50 mL) in a 100 mL round bottom flask equipped with a reflux condenser. The reaction mixture was heated at 60 °C for 24 h. After filtering off the salts, DMF was evaporated and residue was dialysed in MeOH for 3 days to afford product as colourless viscous liquid in 82% yield. ^{1}H NMR (500 MHz, D_2O) δ: 3.42-3.78 (m, PG scaffold's CH and CH_2 protons). IR (neat, cm^{-1}) ν: 3367, 2923, 2874, 2093, 1472, 1457, 1340, 1279, 1066.

Synthesis of $dPG_{10}(N_3)_{0.25}$ and $dPG_{10}(NH_2)_{0.10}$

Azide functionalized dPG was synthesized in two steps, mesylation and substitution by azide following the procedure as reported before.[3] Amine functionalized dPG was synthesized by reducing the $dPG_{10}(N_3)_{0.10}$ using PPh_3 in THF-H2O following the procedure as reported before.[4]

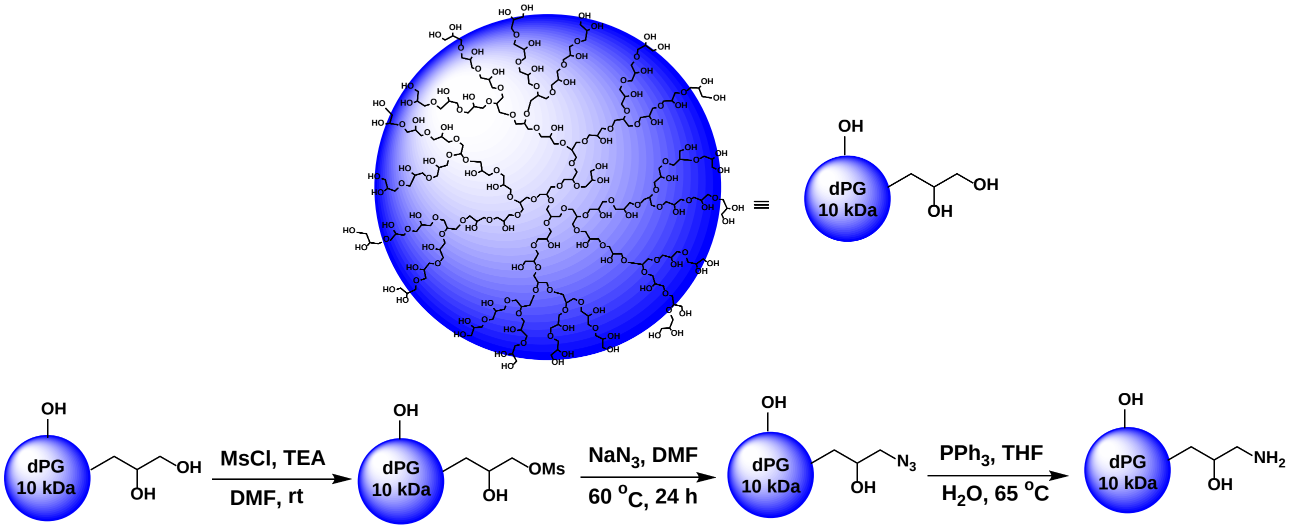

Scheme 2. Synthesis of $dPGN_3$ and $dPGNH_2$ derivatives

Synthesis of Polyglycerol-Sialic acid (PG-SA)

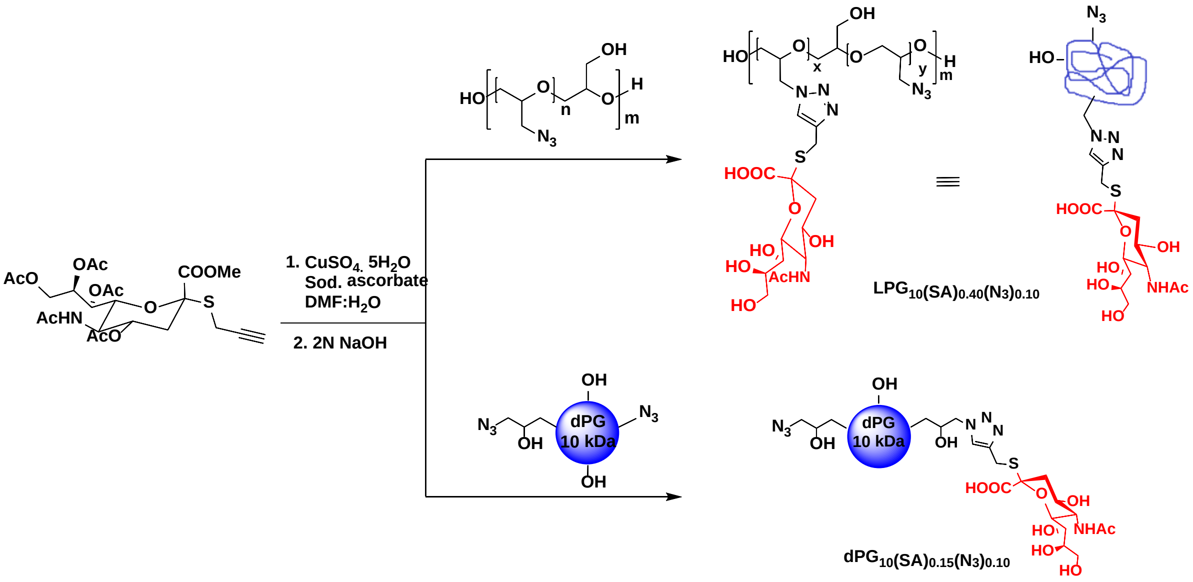

Scheme 3. Synthesis of dPGSA and LPGSA derivatives

Synthesis of $dPG(SA)_{0.15}(N_3)_{0.10}$

dPG-N_3 (DF = 0.25) (100 mg, 0.21 mmol N_3 groups to be functionalized) and prop-2-ynyl α-thiosialoside [127 mg, 0.23 mmol, 1.1 equiv. per azide to be functionalized (15%)] was dissolved in DMF in a round bottom flask. Prop-2-ynyl α-thiosialoside was synthesized using Roy[5] and a slightly modified procedure of Ogura.[6] $CuSO_4.5H_2O$ (23 mg, 0.092 mmol, 0.2 equiv. per azide group to be functionalized) and sodium ascorbate (36 mg, 0.184mmol, 0.4 equiv. per azide to be functionalized) were dissolved in minimum amount of water separately and mixed well to form the yellow colour adduct. This adduct was then added into the DMF solution. Overall reaction mixture was degassed with argon for 10 min. and stirred at 50 ^{o}C for 24 h. After 24 h, DMF was evaporated and residue was stirred in 5 ml 2N aqueous NaOH for 2 h at room temperature. Reaction mixture was then neutralized with 1N HCl and dialysed for 3 days in H_2O and EDTA while changing the solvent thrice a day. Dialysed product was then lyophilized and obtained as white solid in 85% yield. ^{1}H NMR (500 MHz, D2O) δ: 7.93 (bs, 1H, triazolyl proton), 4.55-3.99 (m, 13H), 2.76 (bs, 1H, SA H-3e), 1.99 (s, 1H, NHAc), 1.75 (bs, 1H, SA H-3a). IR (film): ν = 3355, 2922, 2873, 2100, 1609, 1559, 1456, 1388, 1323, 1281, 1250, 1111, 1078, 954 cm^{-1}

Synthesis of $LPG(SA)_{0.40}(N_3)_{0.10}$

LPG-N_3 (DF = 0.50) (100 mg, 1.15 mmol per monomer unit) and prop-2-ynyl α-thiosialoside (277 mg, 0.50 mmol, 1.1 equiv. per azide to be functionalized (40%)) was dissolved in DMF in a round bottom flask. Prop-2-ynyl α-thiosialoside was synthesized using Roy[5] and a slightly modified procedure of Ogura.[6] $CuSO_4.5H_2O$ (23 mg, 0.092 mmol, 0.2 equiv. per azide group to be functionalized) and sodium ascorbate (36 mg, 0.184mmol, 0.4 equiv. per azide to be functionalized) were dissolved in minimum amount of water separately and mixed well to form the yellow colour adduct. This adduct was then added into the DMF solution. Overall reaction mixture was degassed with argon for 10 min. and stirred at 50 ^{o}C for 24 h. After 24 h, DMF was evaporated and residue was stirred in 5 ml 2N aqueous NaOH for 2 h at room temperature.

Reaction mixture was then neutralize with 1N HCl and dialysed for 3 days in H_2O and EDTA while changing the solvent thrice a day. Dialysed product was then lyophilized and obtained as white solid in 82% yield. ^{1}H NMR (500 MHz, D2O) δ: 8.07 (bs, 1H, triazolyl proton), 4.57-3.58 (m, 13H), 2.83 (bs, 1H, SA H-3e), 2.04 (s, 1H, NHAc), 1.87 (bs, 1H, SA H-3a). IR (neat, cm^{-1}) ν: 3280, 2929, 2102, 1716, 1798, 1616, 1557, 1373, 1118, 1029.

Synthesis of Polyglycerol-Bicyclo[6.1.0[non-4-yn-9yl (PG-BCN)

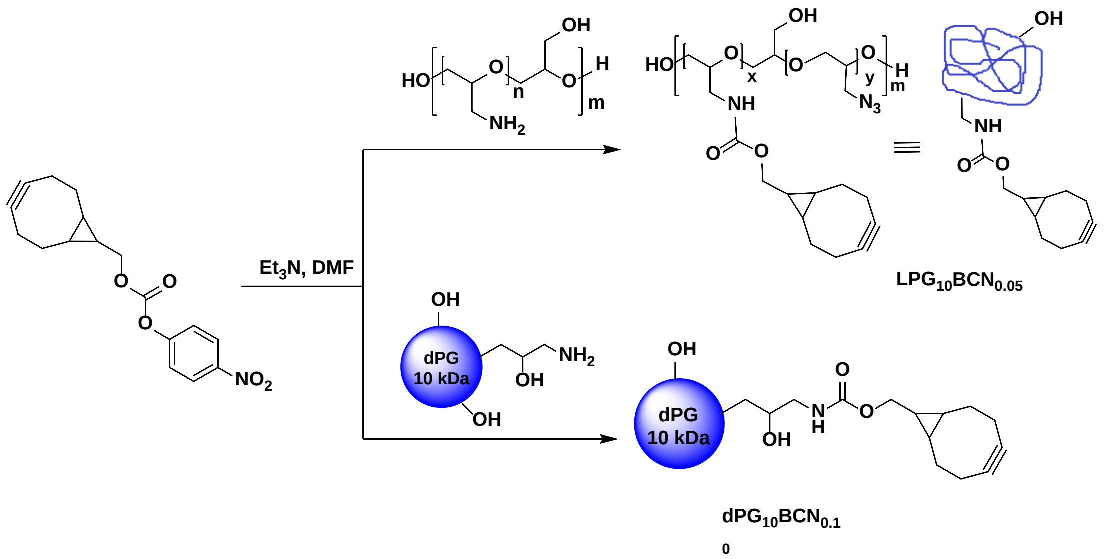

Scheme 4. Synthesis of LPG-(cyclooctyne)$_{5\%}$ and dPG-(cyclooctyne)$_{10\%}$

Synthesis of dPG$_{10}$(cyclooctyne)$_{0.10}$

dPG-NH_2 (DF = 0.10) (1.0 g, 13.5 mmol per monomer unit) was taken in dry DMF (20 ml) along with triethylamine (0. 28 mL, 2.02 mmol, 1.5 equiv. per amino group). Bicyclo[6.1.0[non-4-yn-9ylmethyl (4-nitrophenyl) carbonate (BCN-PNP) (0.64 g, 2.02 mmol, 1.5 equiv. per amino group) was added to the above solution and reaction mixture was stirred for 12 h at room temperature. DMF was evaporated and crude was dialysed in MeOH/H20 (1:1) mixture for 1 day and then in water for 1 more day. The product was stored as a stock solution in H_2O and quantified by lyophilizing a small fraction of aqueous solution. The overall yield was 84% (1.0 g). ^{1}H NMR (500 MHz, D2O) δ: 4.23-3.2 (m, 75H, dPG backbone), 2.46-2.21 (m, 5H), 1.64-1.44 (m, 3H), 1.02-0.82 (m, 3H).

LPG$_{10}$(cyclooctyne)$_{0.05}$

LPG-NH_2 (DF = 5%) (0.5 g, 0.40 mmol NH2, 8 per molecule) was taken in dry DMF (20 ml) along with triethylamine (0.083 mL, 0.6 mmol, 1.5 equiv. per amino group). Bicyclo[6.1.0[non-4-yn-9ylmethyl (4-nitrophenyl) carbonate (BCN-PNP) (0.19 g, 0.6 mmol, 1.5 equiv. per amino group) was added to above solution and reaction mixture was stirred for 12 h at room temperature. DMF was evaporated and crude was dialysed in MeOH/H20 (1:1) mixture for 1 day and then in water for 1 more day. The product was stored as a stock solution in H_2O and quantified by lyophilizing a small fraction of aqueous solution. The overall yield was 88% (0.5 g). δ (ppm) = 4.1-3.1 (m, 70 H, OCOCH_2-oct, LPG backbone), 2.0-2.4 (m, 6H, oct), 1.59–1.22 (br, oct),0.92–0.58 (br, oct)

1.3. Method for nanogel preparation

For the R-NG 1, aqueous solutions of dPG(SA)(N_3) (10 mg, 50 µL, 0.43 µmol) and dPG-cyclooctyne (15 mg, 150 µL, 1.25 µmol) were added in Milli-Q water (5 mL). The solutions were cooled to 4 °C, mixed, and added quickly to magnetically stirred acetone (200 mL) at 900 rpm. The stirring was immediately stopped after addition and the dispersion was left overnight (12 hrs). Then the reaction was quenched by the addition of excess azidoglycerol (1 mmol) dissolved in 1mL of water followed by excess of water (20 mL). Acetone was evaporated to obtain nanogel dispersions in water. The nanogels were dialyzed in water for 2 days and concentrated by evaporation of water. Concentration was determined by lyophilizing a small part of the solution. Nanogels were stored as highly concentrated stock solutions in water in the fridge below 20 °C. The other F-NGs nanogels were prepared using the same procedure (Table 1). For the preparation of C-NG (without SA), macromonomers dPG$(N_3)_{10\%}$ (10 mg, 50 µL, 1.0 µmol) and dPG-(cyclooctyne)$_{10\%}$ (15 mg, 150 µL, 1.25 µmol) were used and the rest of the procedure remained same as for the R-NG 1.

1.4. Cryo-Transmission Electron Microscopy (Cryo-TEM)

Perforated carbon film-covered microscopical 200 mesh grids (R1/4 batch of Quantifoil, MicroTools GmbH, Jena, Germany) were cleaned with chloroform and hydrophilised by 60 s glow discharging at 8 W in a BAL-TEC MED 020 device (Leica Microsystems, Wetzlar, Germany) before 5 µl aliquots of the corresponding sample solution were applied to the grids. The samples were vitrified by automatic blotting and plunge freezing with a FEI Vitrobot Mark IV (ThermoFisher Scientific Inc, Waltham, USA) using liquid ethane as cryogen. The vitrified specimens were transferred subsequently under liquid nitrogen into a FEI Tecnai F20 TEM (ThermoFisher Scientific Inc, Waltham, USA) equipped with a field emission gun and operating at 160 kV or into a FEI TALOS ARCTICA TEM (Thermo Fisher Scientific, Waltham, USA), equipped with a high-brightness field-emission gun (XFEG) operated at an acceleration voltage of 200 kV. Microscopy was carried out at a 94 K sample temperature using the low-dose protocol of the microscopes. Micrographs were acquired on an FEI Eagle 4k × 4k CCD camera using a twofold binning mode (on the Tecnai F20) or on a FEI Falcon 3 direct electron detector (on the Talos Arctica).

To generate stereo images, the same image section was recorded at two different tilt angles (4° and -4°) using the compuStage of the corresponding microscope. The resulting micrographs were aligned using the software StereoPhoto Maker, Version 4.41 (Masuji Suto, Japan).

For binding experiments nanogel samples (~1 mg/ml in PBS, pH 7.4) were incubated with influenza virus A/X31(H3N2) (~1 mg/ml total protein) with gentle agitation for 30 min at room temperature prior to the cryo-preparation.

Cryo-electron tomography (Cryo-ET)

Tomograms were acquired on a FEI TALOS ARCTICA transmission electron microscope (ThermoFisher Scientific Inc., Waltham (MA), USA) operating at 200 kV. Single axis tilt series (±64° in 2° tilt angle increments) were recorded with a Falcon-II 4k × 4k direct electron detector at full resolution (29 K primary magnification) with a total dose less than 70 e-/Å2. Tomogram reconstruction was done using ThermoFisher Inspect3D software, Version 3.1.0.

1.5. AFM measurement

An atomic force microscope Multimode 8 with a Nanoscope V controller (Veeco Instruments, Santa Barbara, California, USA) and equipped with a closed fluid chamber was used for all measurements. Cleaved mica coated with a layer of Poly-L-lysine (Sigma-Aldrich MW: 70-150kDa) was used as a substrate for better attachment of nanogels on the surface during imaging. A 10 µl solution was deposited at the center of freshly cleaved mica and allowed to dry for at least 15 minutes. Afterwards, the mica surface was rinsed repeatedly with Milli-Q and allowed to dry again. Aqueous sample solutions of concentration 1mg /mL were 10-fold diluted in Milli-Q and 5 µl sample were deposited at the center of the mica discs and allowed to rest for 10 minutes at room temperature. After that, the samples were gently rinsed and a thin layer of liquid remained. The sample was never allowed to dry. Afterwards, the sample was mounted on the head of the AFM, where the fluid chamber was assembled and allowed to thermally reach equilibrium for about 15 minutes.

Cantilever A, type SNL-10 from Bruker with nominal tip radius of 2 nm and nominal spring constant 0.3N/m were used in these experiments. Prior imaging, cantilevers were calibrated on hard mica surface. A compression on mica was taken to extract the cantilever sensitivity and then, the thermal noise method was used to obtain its spring constant. Imaging in PeakForce mode was carried out with a maximal applied force of 500 pN using a 512 points per line at a scan rate of 0.7Hz. These imaging conditions were stable enough to repeatedly image the particles without causing any serious structural damage. The data analysis was done using NanoScope Analysis 1.4 software.

1.6. TIRF measurement

Total internal reflection fluorescence (TIRF) microscopy was used to quantify the specific interaction of single, R18-labelled IAVs viruses with its native attachment factor, sialic acid, embedded in a supported lipid bilayer (SLB) as described in literature.[7] This method provides information about various properties of the IAV-sialic acid interaction, such as the IAV attachment rate to sialic acid-presenting SLB and how this attachment rate is modified upon addition of virus binding inhibitors. Sialic acid containing GD1a gangliosides (2.5 wt.%, 1 mol%) were incorporated in phospholipid liposomes containing POPC (96.2 wt.%, 1-palmitoyl-2-oleoyl-glycero-3-phosphocholine) and DSPE-PEG2k (3.8 wt.%, 1 mol%, 1,2-distearoyl-sn-glycero-3-phosphoethanolamine-N-[carboxy(polyethyleneglycol)-2000] (sodium salt)) and GD1a-containing SLBs were formed using the vesicles rupture method. Videos were taken to visualize the transient binding of IAV to the GD1a-containing SLB, allowing for extracting the rate of IAV attachment to the SLLB.[7,8,9] For sufficiently larger inhibitor concentrations, a strong decrease in attachment rate is typically observed, which allows for determining an inhibition concentration (IC_{50} value) based on the capability of the inhibitor to prevent IAV attachment to attachment factor-presenting lipid membranes. These measurements have been conducted as follows:

Materials: Ganglioside GD1a (disialoganglioside-GD1a (porcine brain, diammonium salt)), POPC (1-palmitoyl-2-oleoyl-glycero-3-phosphocholine), DSPE-PEG2k carboxylic acid (1,2-distearoyl-sn-glycero-3-phosphoethanolamine-N-[carboxy(polyethylene glycol)-2000] (sodium salt)), were obtained from Avanti Polar Lipids Inc. (Alabaster, AL). Potassium

chloride (>99.5 %) was obtained from Grüssing (Filsum, Germany). Tris-HCl (tris(hydroxymethyl)aminomethane hydrochloride), R18 (octadecyl rhodamine B chloride), calcium chloride (≥97 %), and liquinox cleaning solution were purchased from Sigma Aldrich (Steinheim, Germany).

SLB formation: Vesicles (0.33 mg/mL) composed out of POPC (96.2 wt.%), GD1a ganglioside (2.5 wt.%, 1 mol %) and DSPE-PEG2k carboxylic acid (3.8 wt.%, 1 mol%) in tris-HCl buffer (0.1 M tris-HCl, 50 mM potassium chloride, 5 mM calcium chloride; pH 7.4 adjusted with HCl) were produced by small volume extrusion. Glass slides (cover slips, 25 mm) were cleaned in a 0.5 % aqueous solution of Liquinox in a high beaker (250 mL) at 80 °C for 75 min. The glass slides were rinsed intensively with milli-Q water and dried at room temperature before a home-made polydimethylsiloxane (PDMS) well was placed on the glass and the vesicle solution added to each well. The SLB is formed by adsorption and rupturing at high densities of the vesicles on the glass surface.[10] Non-adsorbed vesicles were washed away with replacing the supernatant 10 times with PBS (pH 7.4).

IAV labelling: Influenza A/X31 (H3N2) viruses propagated in embryonated chicken eggs for 48 h were purified with ultracentrifugation at 100 000 x g. BCS assay was used to determine a total protein content of 3.0 mg/mL. Aliquots of 5 µL were stored at -80 °C. 5 µL X31 solution was mixed with 93 µL PBS buffer and 2 µL R18 fluorescent dye (0.2 mM in ethanol). The mixture was stored for 30 min on ice. Afterwards, non-membrane-incorporated R18 dye was removed with a microspin column PD SpinTrap G-25 (2 min, 3300 rpm, GE Healthcare, Buckinghamshire, UK). After centrifugation the virus solution was diluted with 400 µL PBS buffer. The virus solution was homogenized 1 to 1 (v/v) with sialylated nanogel solution and applied in the well on top of the SLB.

TIRF microscopy: An Eclipse Ti-E (Nikon, Tokyo, Japan) microscope equipped with a 100x oil objective (NA XXX, Nikon), a Zyla 4.2 sCMOS camera (Andor, Oxford, UK) and a white light source (Lumen 200; Prior Scientific, Cambridge, UK) was used to record videos of the virus interaction with the receptors within the SLB and the impact of the binding due to the addition of sialylated nanogels. Five TIRF videos with 1000 frames (acquisition rate 9 frames per second, exposure time 80 ms, 2 x 2 binning, field of view 130 µm x 130 µm, pixel size 130 nm x 130 nm) were recorded for each measurement condition to quantify the transient interaction of IAVs with the SLB. The IAV attachment rate was determined using single virus tracking implemented by home-made MATLAB scripts as described in literature.[7]

1.7. Biological assays

Virus

All experiments were done using influenza virus A virus X31 strain (A/Aichi/2/68, H3N2) which is reassorted with segments of A/PuertoRico/8/1934 H1N1. Virus was grown in embryonated chicken eggs. After four days of incubation allantoic fluid were harvested and separated from fragments by centrifugation (2500xg, 15 min). This suspension was used for infection inhibition assays. Viruses used for hemagglutination inhibition assays were further purified and enriched by ultra-centrifugation (100,000g, 1,5h). Viruses were either produced in own facilities or were kindly supplied by Thorsten Wolff (Robert-Koch-Institute Berlin).

Cell culture

Infection inhibition studies were done using MDCK-II (Madin-Darby canine kidney epithelial) cells originally purchased from ATCC. Cells were grown using DMEM (supplemented with 10% fetal bovine serum, 2mM L-glutamine, 100µg/ml streptomycin and 100units/ml penicillin) at 37 °C and 5% CO_2. All solutions were purchased from PAN Biotech.

Hemagglutination Inhibition Assay

The assay was performed based on previous published protocols.[3] Twofold dilution series of the inhibitor compound in PBS in a total volume of 20µl were done in a 96 well dish, round bottom. Afterwards, 5µl virus was added to each dilution. Each droplet of 5µl corresponds to 4 HA units (4 x 10^7 virus particles) which was estimated as published by Desselberger.[11] NGs suspension was incubated for 30 minutes under slight agitation at RT. Afterwards 50µl of a 1% human red blood cell suspension (2 x 10^6 cells per µl, blood was purchased from German Red Cross) were added to the NG-virus complexes, carefully mixed and incubated for another hour at RT without any agitation. Incubation for 1 hour at RT is followed by determining the $K_{i(HAI)}$ values. Here, the last well showing agglutination represents the lowest effective inhibitor concentration.

Cell Toxicity Assay

To determine if the NGs affect cell proliferation, cell growth was measured using the CellTiter 96® Aqueous One Solution Cell Proliferation Assay (Promega). The calorimetric assay is based on the MTS salt [3-(4,5-dimethyl-2-yl)-5-(3-carboxymethoxyphenyl)-2-(4-sulfophenyl)-2H-tetrazolium] which becomes reduced according to the available amount of NADPH or NADH to formazan, which itself can be detected by absorbance at 490nm.

To measure the effect of the NGs on MDCK-II proliferation the medium was exchanged with a DMEM based medium containing inhibitors at a concentration of 250µg/ml. Under these conditions, cells were grown for 2 days. Afterwards the number of viable cells was determined using the CellTiter 96® Aqueous One Solution Cell Proliferation Assay. Cells were incubated with Aqueous One Solution for 75 to 90 minutes. To unravel any toxic effect of NGs cells were incubated with or without NGs at different concentrations in the absence of viruses.

Infection Inhibition Assay

To test whether the compounds can inhibit cell infection by X31 virus 20.000 MDCK-II cells were seeded in a 96-well dish, flat bottom one day before infection. On day of infection first compounds were either diluted in a twofold or tenfold serial dilution series in PBS++ with a total volume of 45µl. Virus were added that each well corresponds to a MOI of 0.5 and incubated for 30 minutes at RT under slight agitation. Cells were washed once with PBS++ followed by treatment with the compound-virus suspension for one hour at RT without any agitation. Medium on the cells was removed, cells were washed once with infection media (DMEM supplemented with 0.2% fetal bovine serum, 0.2% bovine serum albumin, 2.5µg/ml TPCK Trypsin, 2mM L-glutamine, 100µg/ml streptomycin and 100units/ml penicillin; all solutions were purchased from PAN Biotech) followed by covering the cells with 100µl of fresh

infection media. After 2 days of incubation at 37 °C and 5% CO_2 the number of viable cells was determined using again the assay (CellTiter 96® Aqueous One Solution Cell Proliferation Assay (Promega)) as described before. Again, incubation with Aq_{ueous} One Solution was done for 75 to 90mins. Results were normalized to the maximal cell death caused by X31 infection using equation (1)

$$cell\ viability\ (\%) = \frac{(infected_{treated} - infected_{untreated})}{(uninfected_{untreated} - infected_{untreated})}$$

Every data point of the dose response curve represents a mean values (n≥6). To obtain the IC_{50} value data were fitted using a four parametric logistic fit.

1.8 Confocal studies for virus blocking The virions of A/X31 (H3N2) were labelled with 3,3-Dioctadecyloxacarbocyanine perchlorate (DiO). 5 µL 20 µM DiO (ethanol) were added to 100 µL A/X31 (H3N2) suspension (protein content: 0.36 mg/mL, particle content: 2.3×10^9 particles/mL). After incubation for 45 min in dark, the free dye was removed by spinning column (Protein A HP SpinTrap™, GE Healthcare, Germany). Afterwards, 10 µL DiO labelled virion was incubated with 90 µL of 100 µg/mL inhibitor suspension for 45 min. The mixture was then applied to MDCK-II cells and incubated for 2 hours at 20 ^oC. Unbound virions were removed by washing with PBS three times. The cell nucleus was stained with Hoechst 23358 and the cells were visualized by confocal laser scanning microscopy (Leica SP8, Germany).
ImageJ software was used for the image analysis. For viral particle counting, the image was converted to 8-bit grey-scale format and then the detection threshold was selected accordingly (in this study, 14-255) to remove the background. Afterwards, the viral particles were analysed via 'particle analysis' and counted by comparing the pixel size to single viral particles. The inhibition was calculated as following:

$$Inhibtion\ (\%) = \left(1 - \frac{Virus\ density\ (sample)}{Virus\ density\ (control)}\right) \times 100\%$$

2. Supporting Figures and Tables

2.1. ^{1}H NMR of compounds

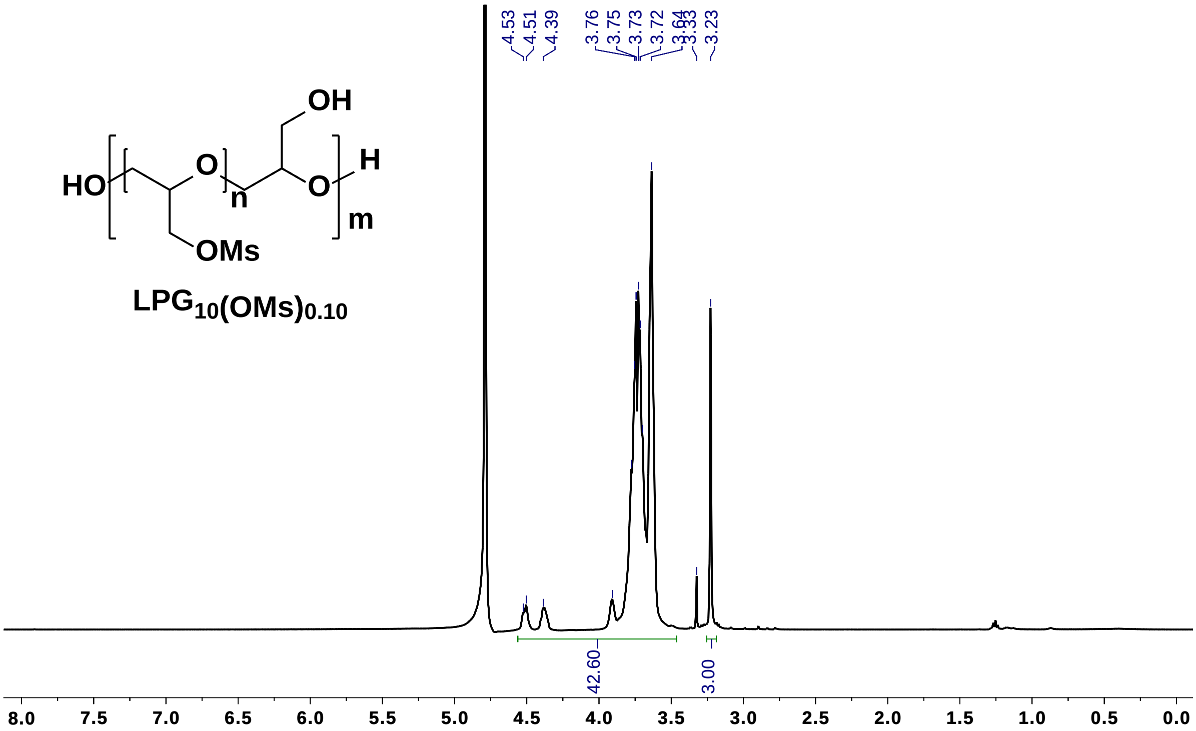

Figure S1. ^{1}H NMR of $LPG_{10}(OMs)_{0.10}$ in D_2O at 500 MHz.

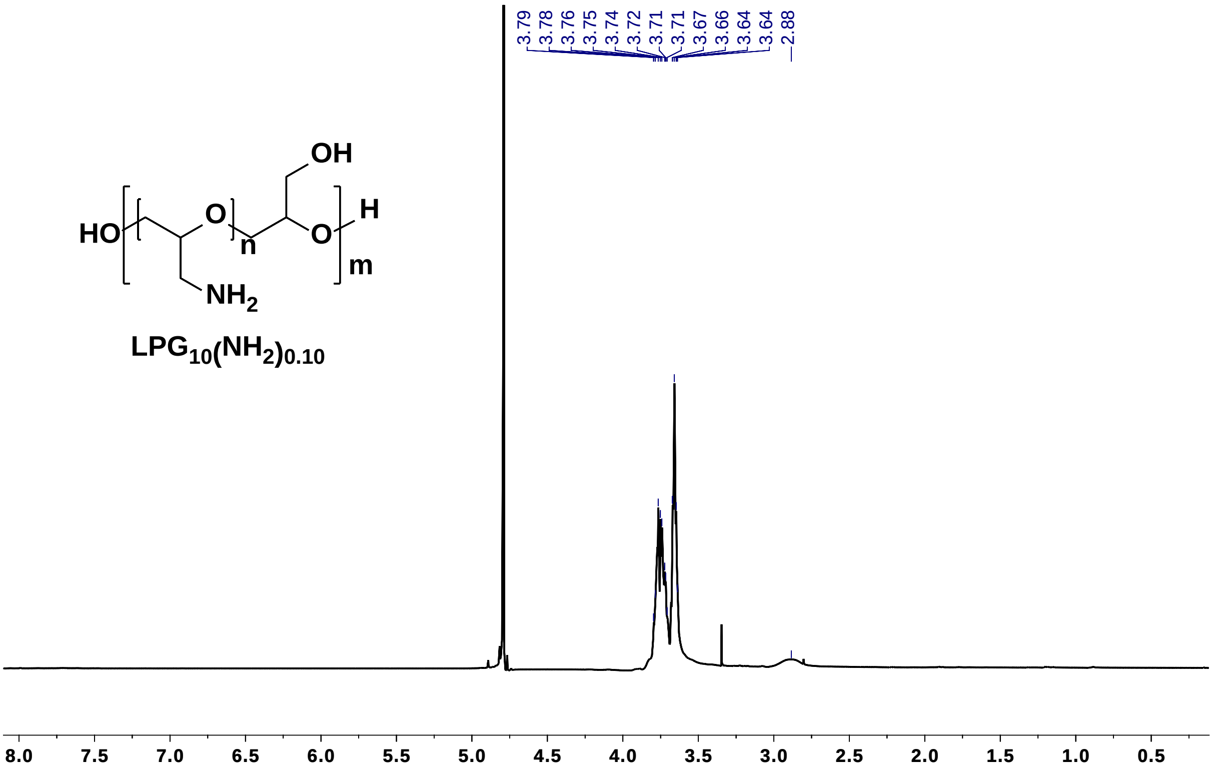

Figure S2. ^{1}H NMR of $LPG_{10}(NH_2)_{0.10}$ in D_2O at 500 MHz.

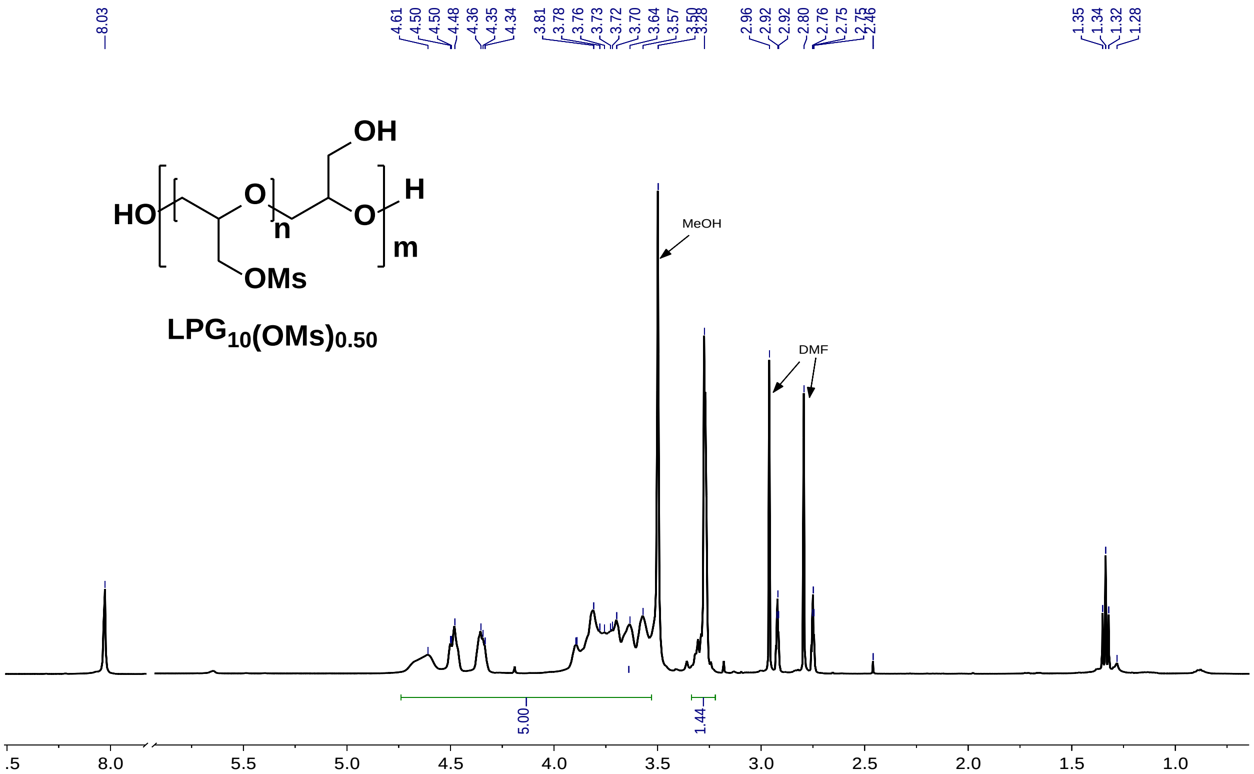

Figure S3. ^{1}H NMR of $LPG_{10}(OMs)_{0.50}$ in DMF-d_7 at 500 MHz.

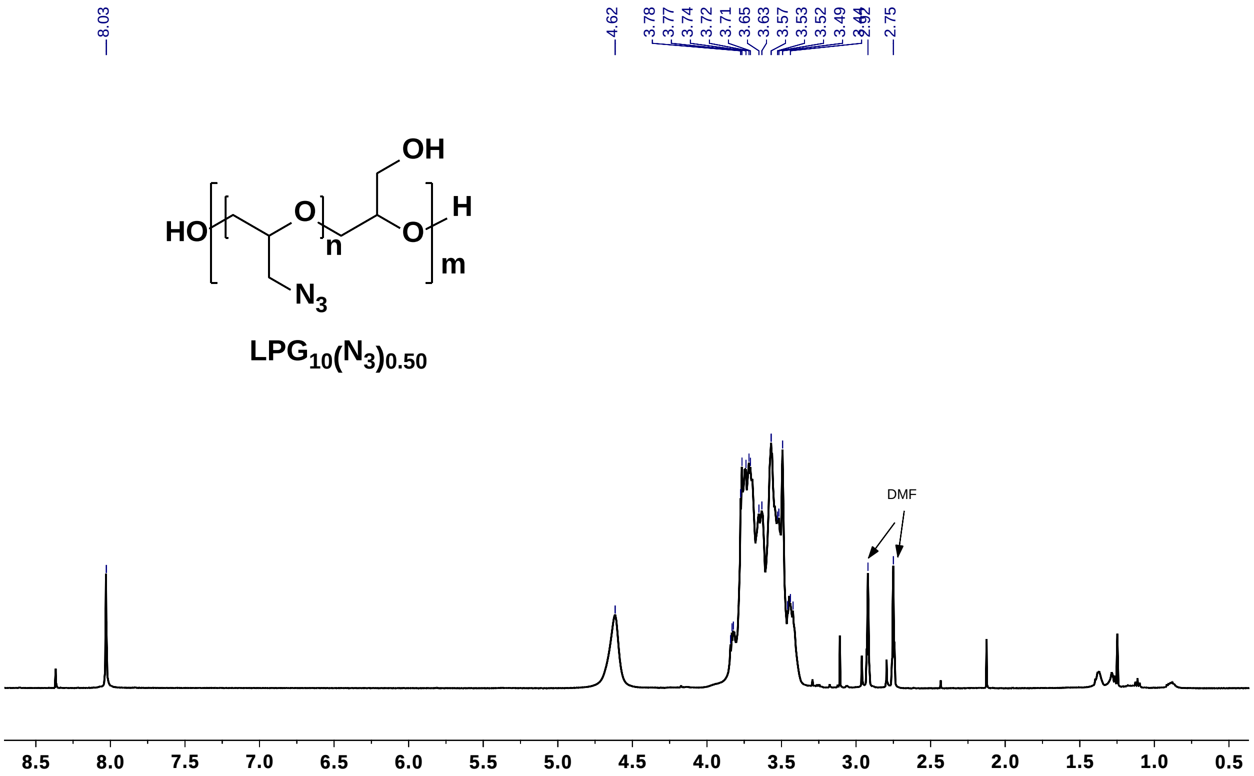

Figure S4. ^{1}H NMR of $LPG_{10}(N_3)_{0.50}$ in DMF-d_7 at 500 MHz.

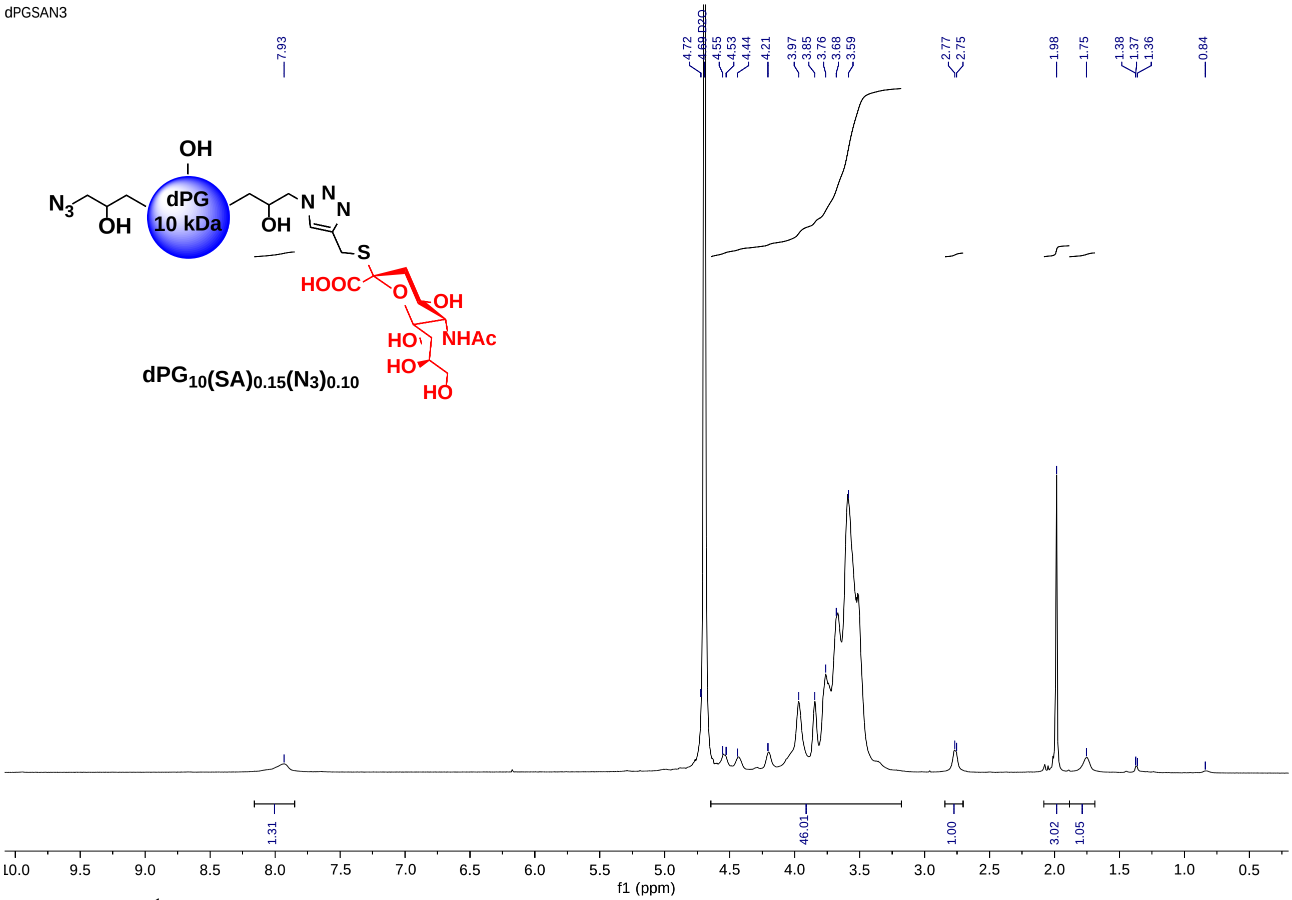

Figure S5. ^{1}H NMR of dPG$(SA)_{0.15}(N_3)_{0.10}$ in D_2O at 500 MHz.

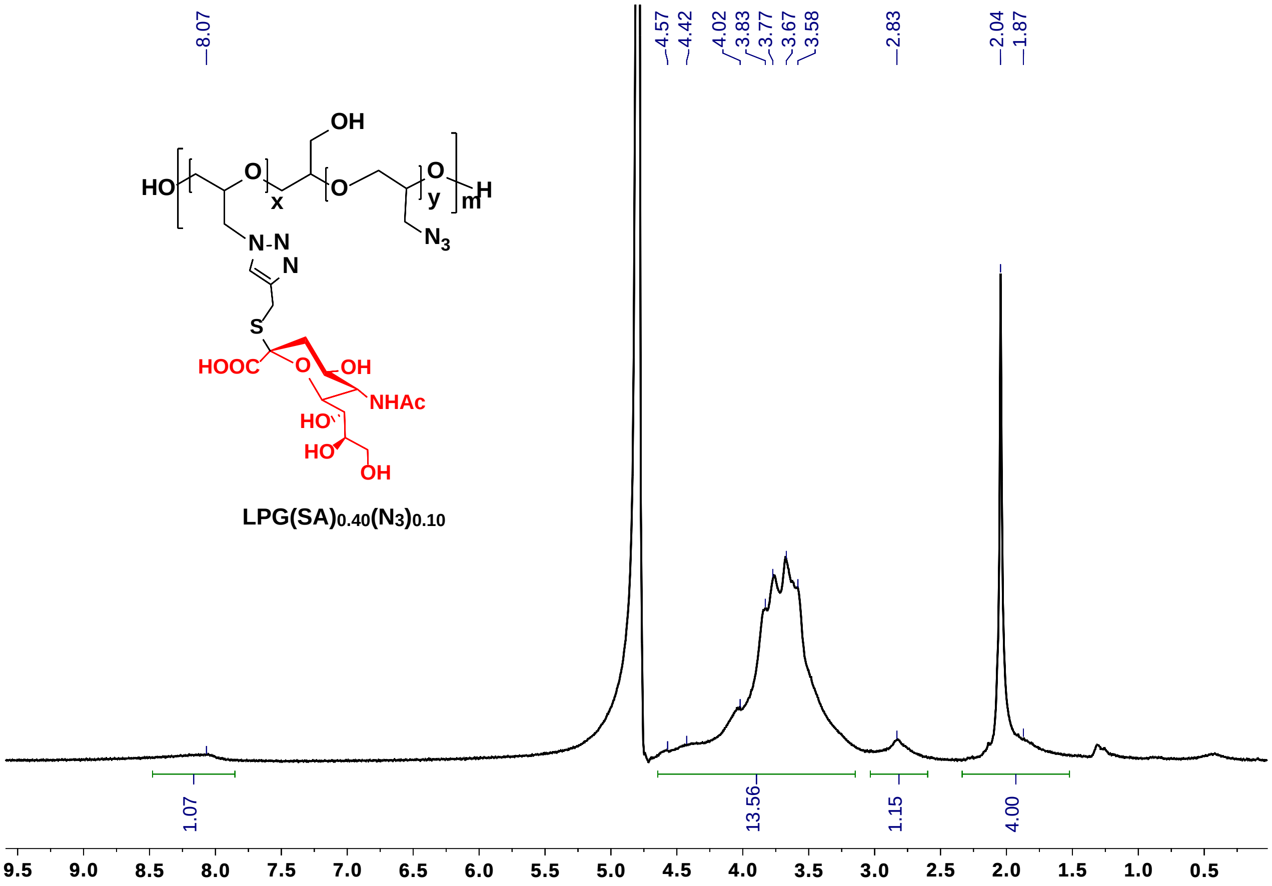

Figure S6. ^{1}H NMR of LPG$(SA)_{0.40}(N_3)_{0.10}$ in D_2O at 500 MHz.

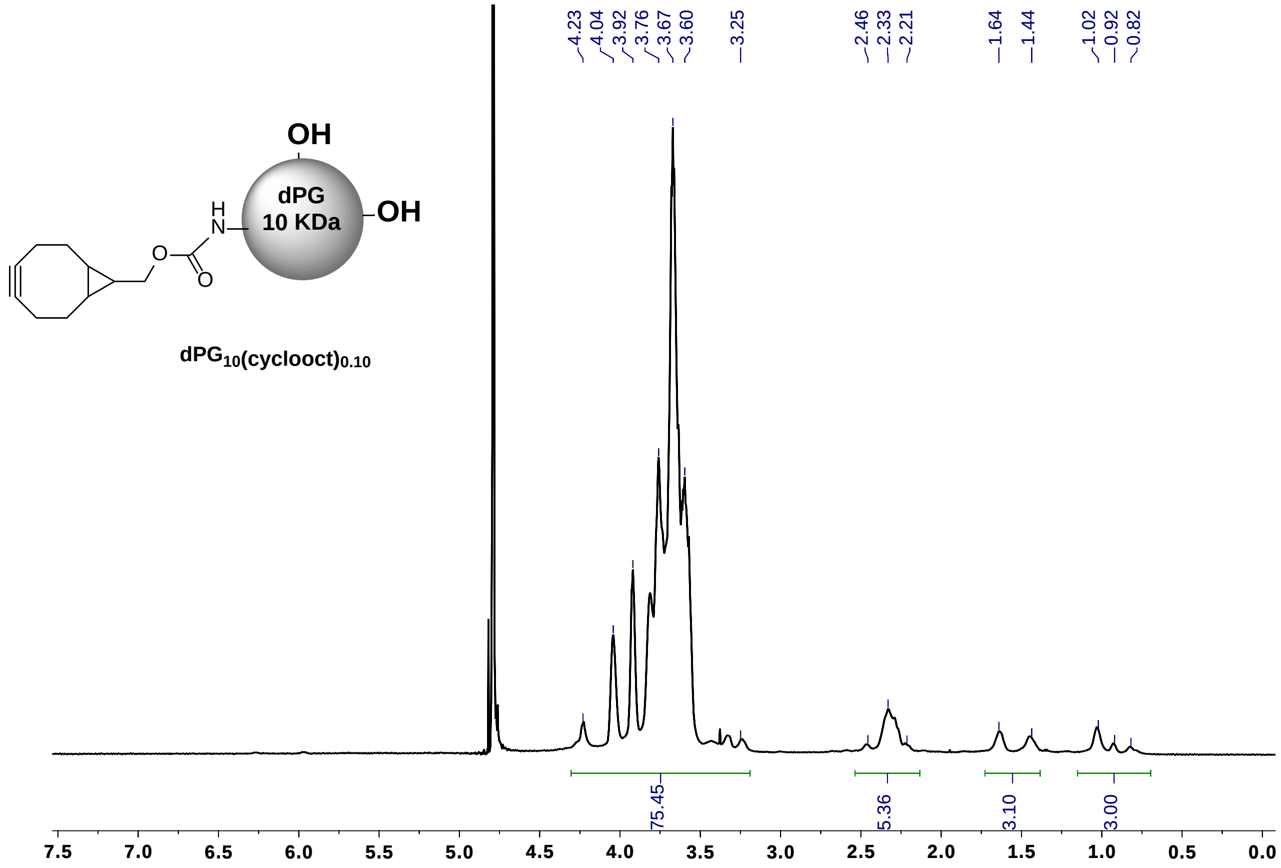

Figure S7. ^{1}H NMR of $dPG_{10}BCN_{0.10}$ in D_2O at 500 MHz.

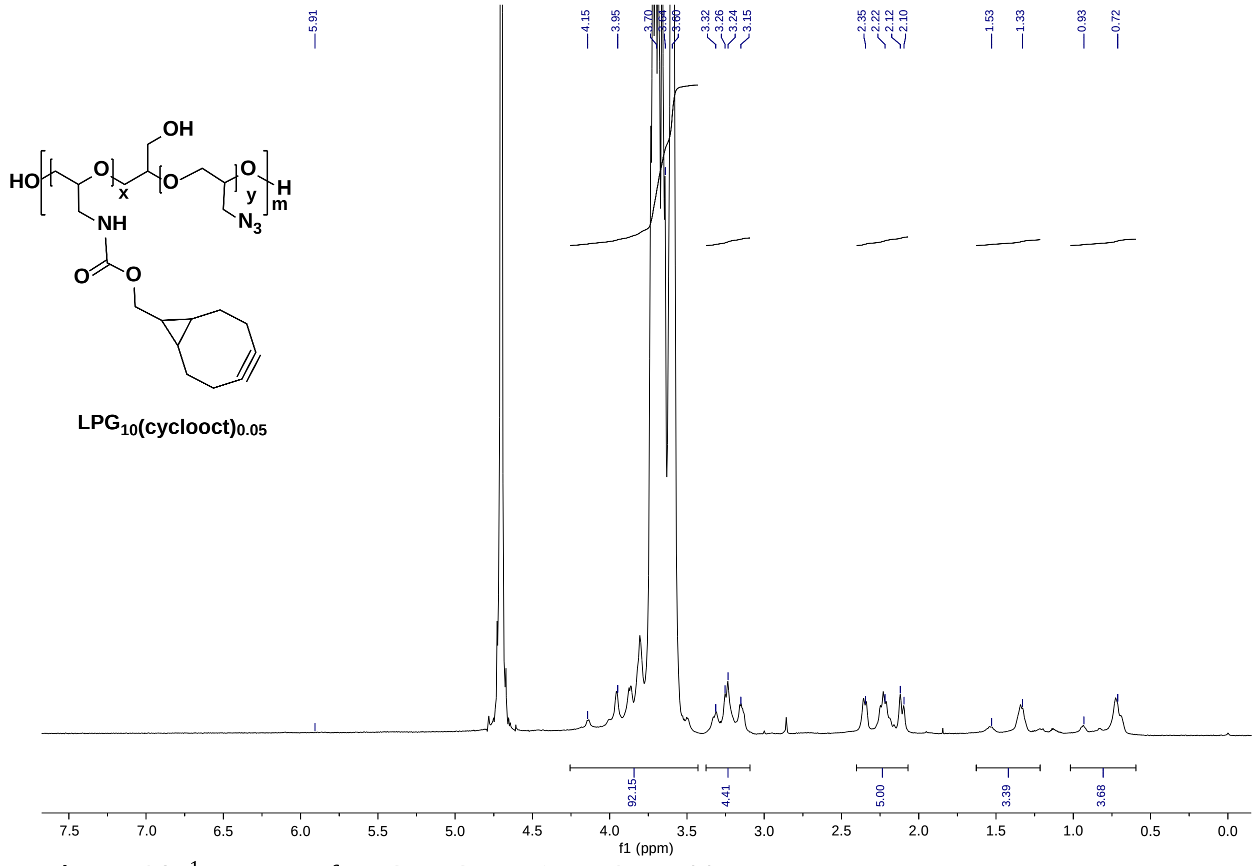

Figure S8. ^{1}H NMR of $LPG_{10}BCN_{0.05}$ in D_2O at 700 MHz.

^{1}H NMR of nanogels

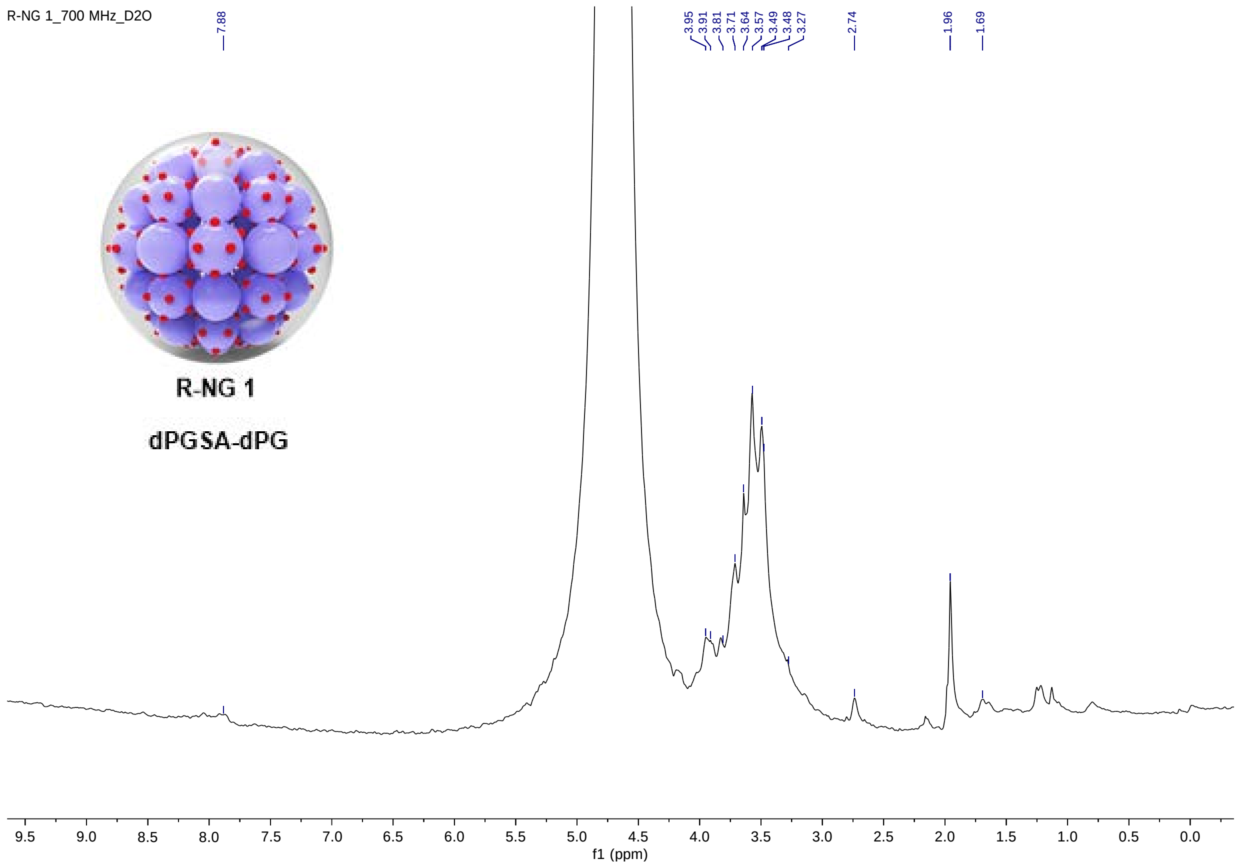

Figure S9. ^{1}HNMR of R-NG 1 at 700 MHz in D_2O.

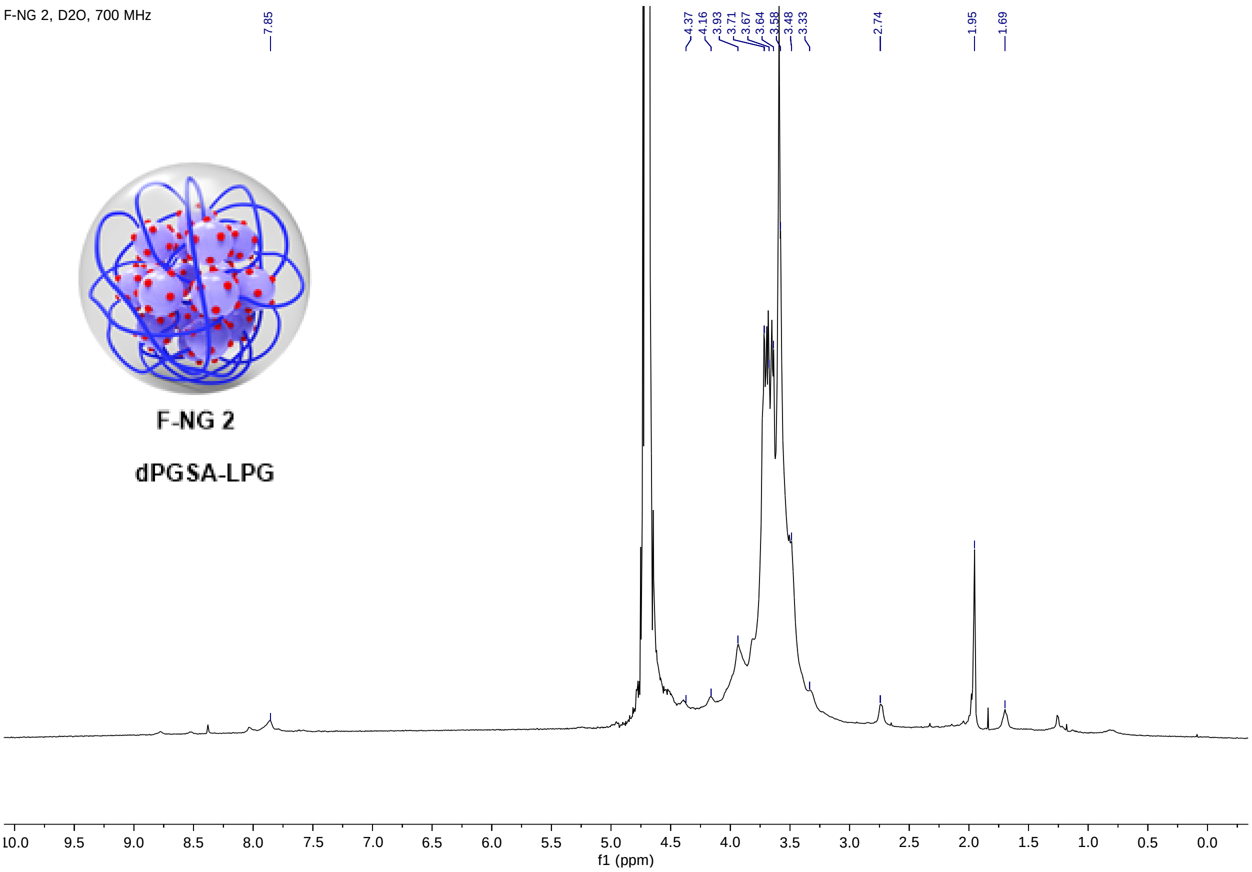

Figure S10. ^{1}HNMR of F-NG 2 at 700 MHz in D_2O.

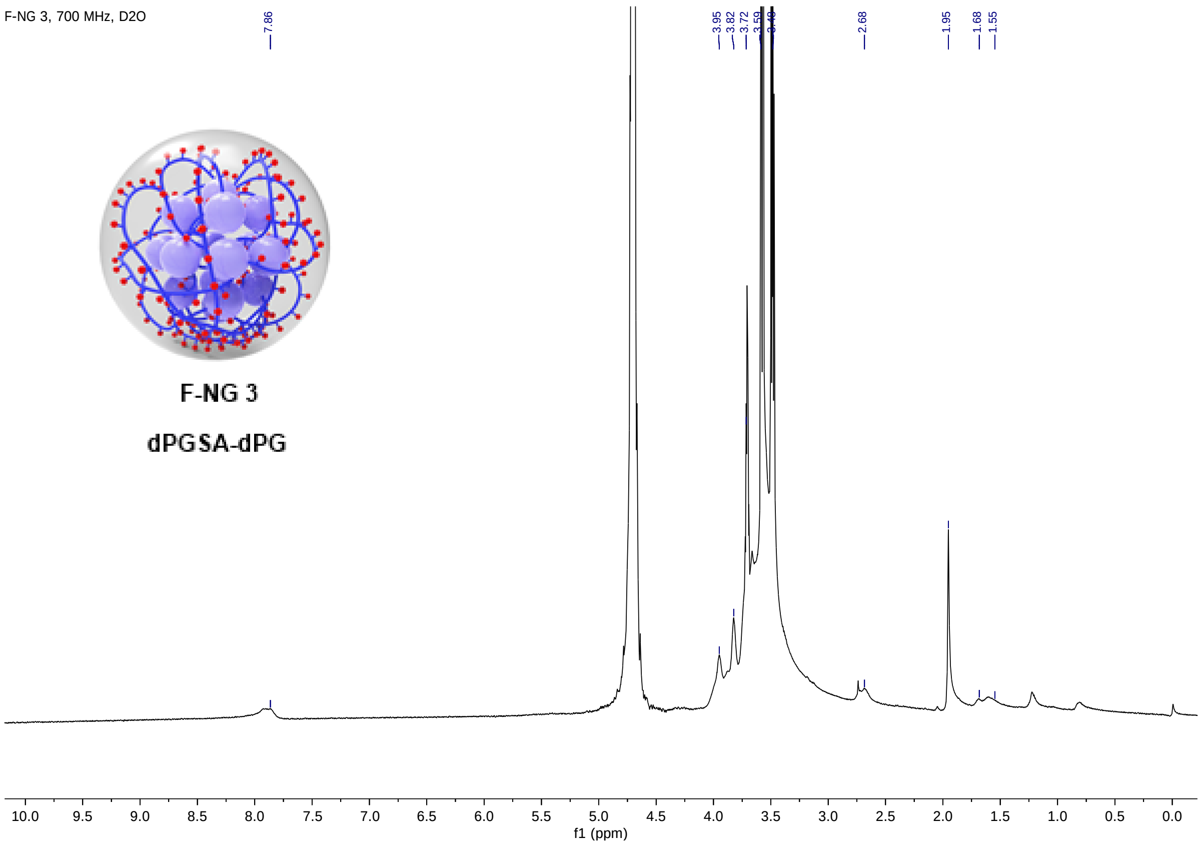

Figure S11. ^{1}HNMR of F-NG 3 at 700 MHz in D_2O.

2.2. Size distribution profiles by DLS

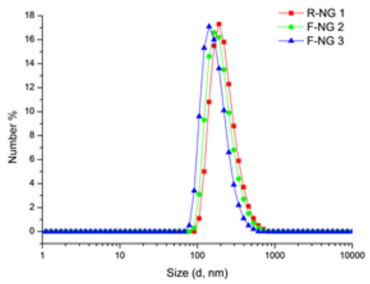

Figure S12. Number distribution profile of R-NG 1, F-NG 2, and F-NG 3 in PBS at concentration of 1 mg/mL.

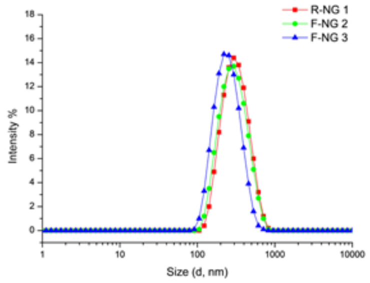

Figure S13. Intensity distribution profile of R-NG 1, F-NG 2, and F-NG 3 in PBS at concentration of 1 mg/mL.

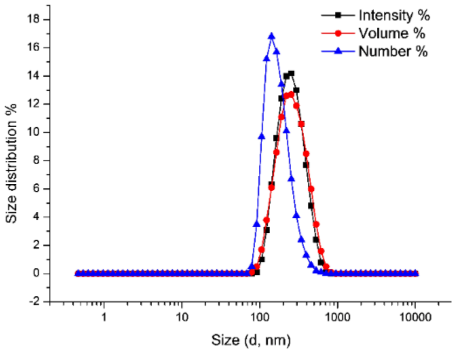

Figure S14. Size distribution profile of control nanogel (C-NG) without sialic acid in H_2O at concentration of 0.5 mg/mL. Z-Ave (d, nm) = 230.6 ± 1.8, PDI = 0.13

2.3. Cryo-TEM of nanogels with influenza A virus

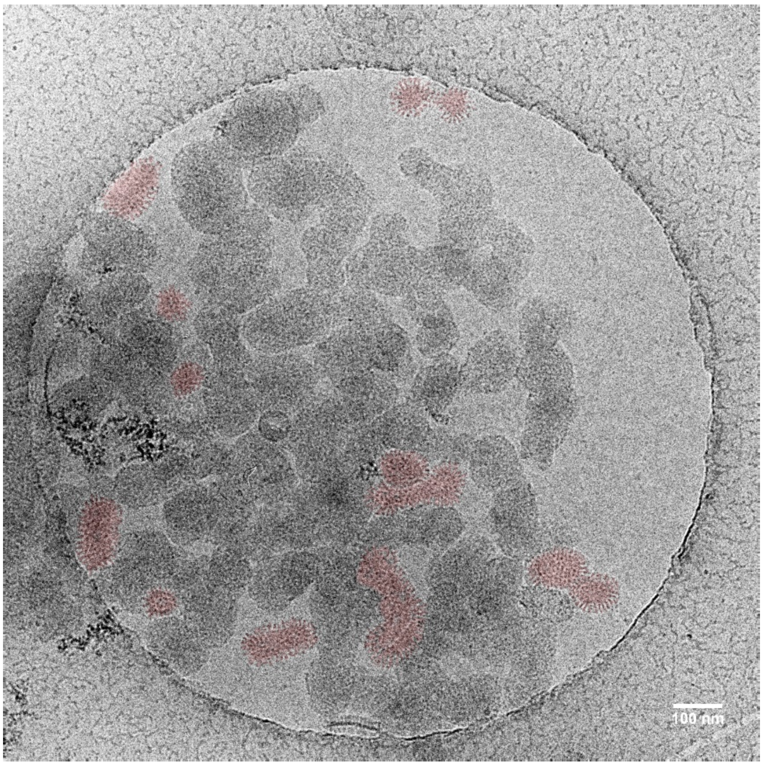

Figure S15. Cryo-electron micrograph of R-NG 1 incubated with IAV X31 in PBS pH 7.4 for 30 min at room temperature and embedded in vitreous ice. The virus particles are colored red for better recognition.

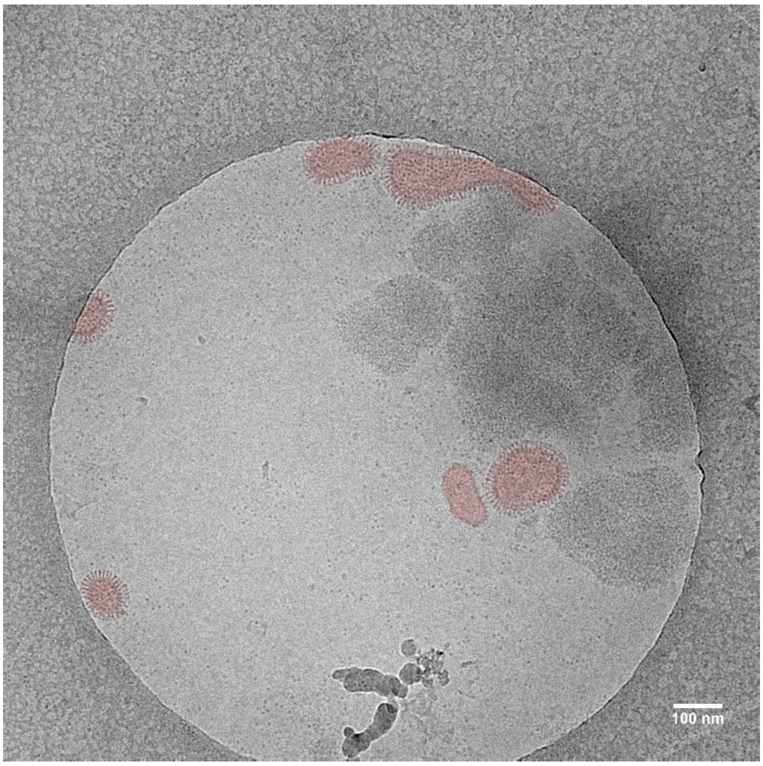

Figure S16. Cryo-electron micrograph of F-NG 2 incubated with IAV X31 in PBS pH 7.4 for 30 min at room temperature and embedded in vitreous ice. The virus particles are colored red for better recognition.

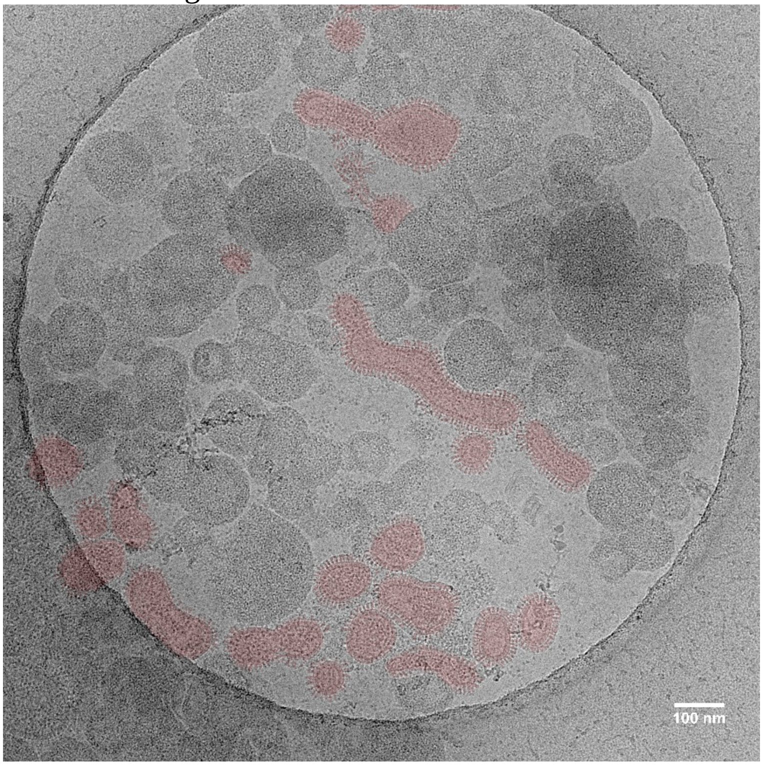

Figure S17. Cryo-electron micrograph of F-NG 3 incubated with IAV X31 in PBS pH 7.4 for 30 min at room temperature and embedded in vitreous ice. The virus particles are colored red for better recognition.

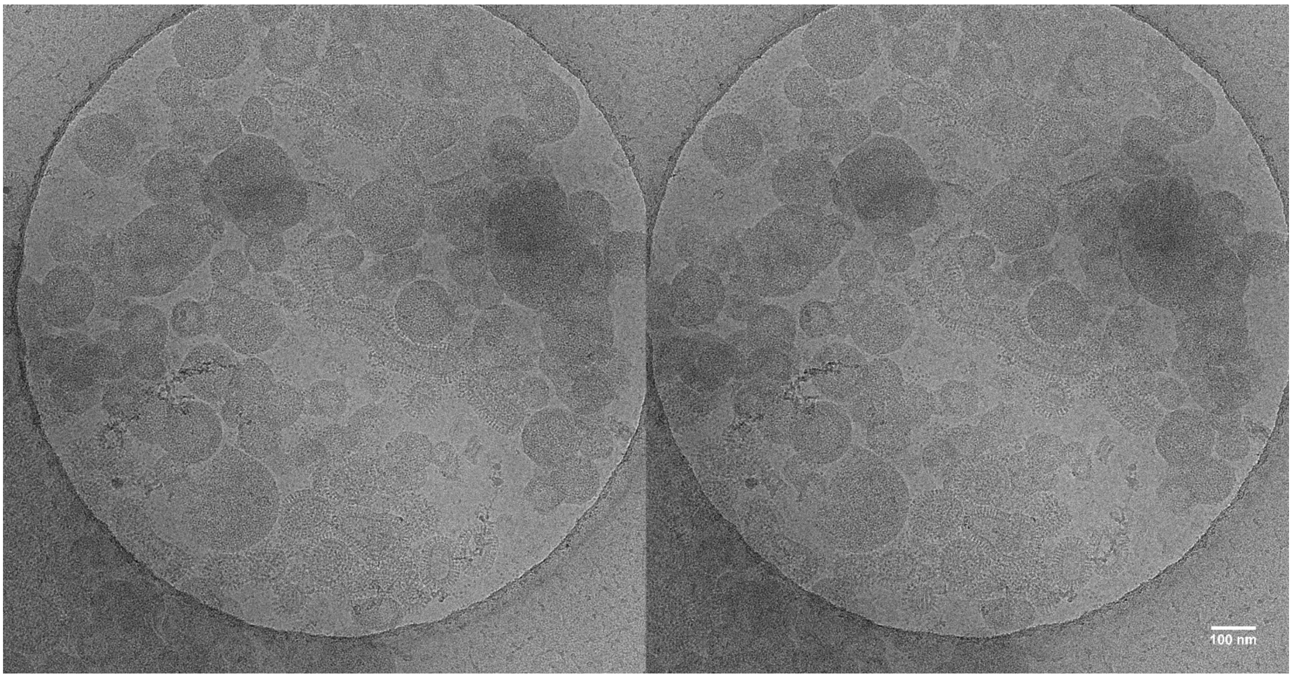

Figure S18. Example of a stereo image (shown as side-by-side stereogram) of F-NG 3 incubated with IAV X31 in PBS pH 7.4 for 30 min at room temperature and embedded in vitreous ice. This stereogram corresponds to the micrograph shown in Figure S6.

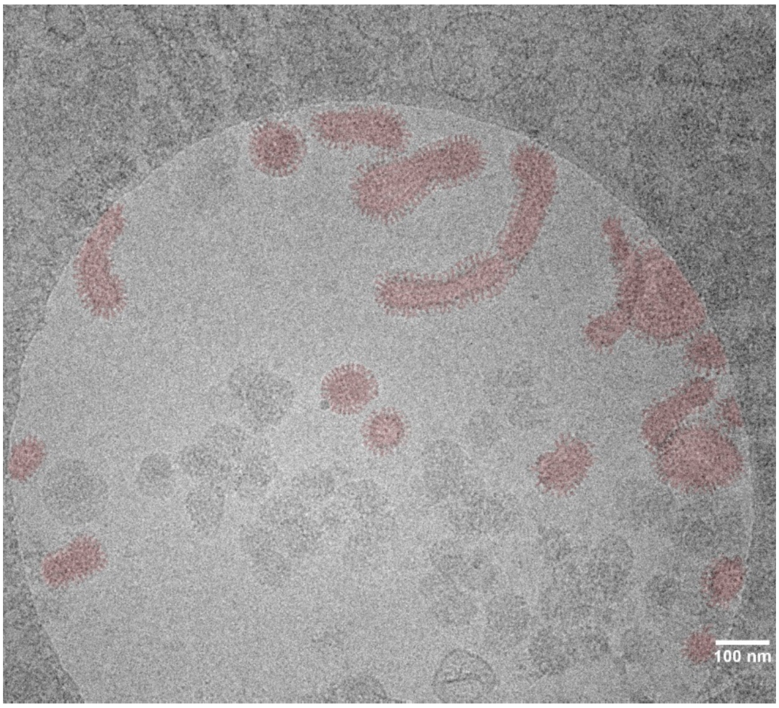

Figure S19. Example of Control-NG incubated with IAV X31 in PBS pH 7.4 for 30 min at room temperature and embedded in vitreous ice.

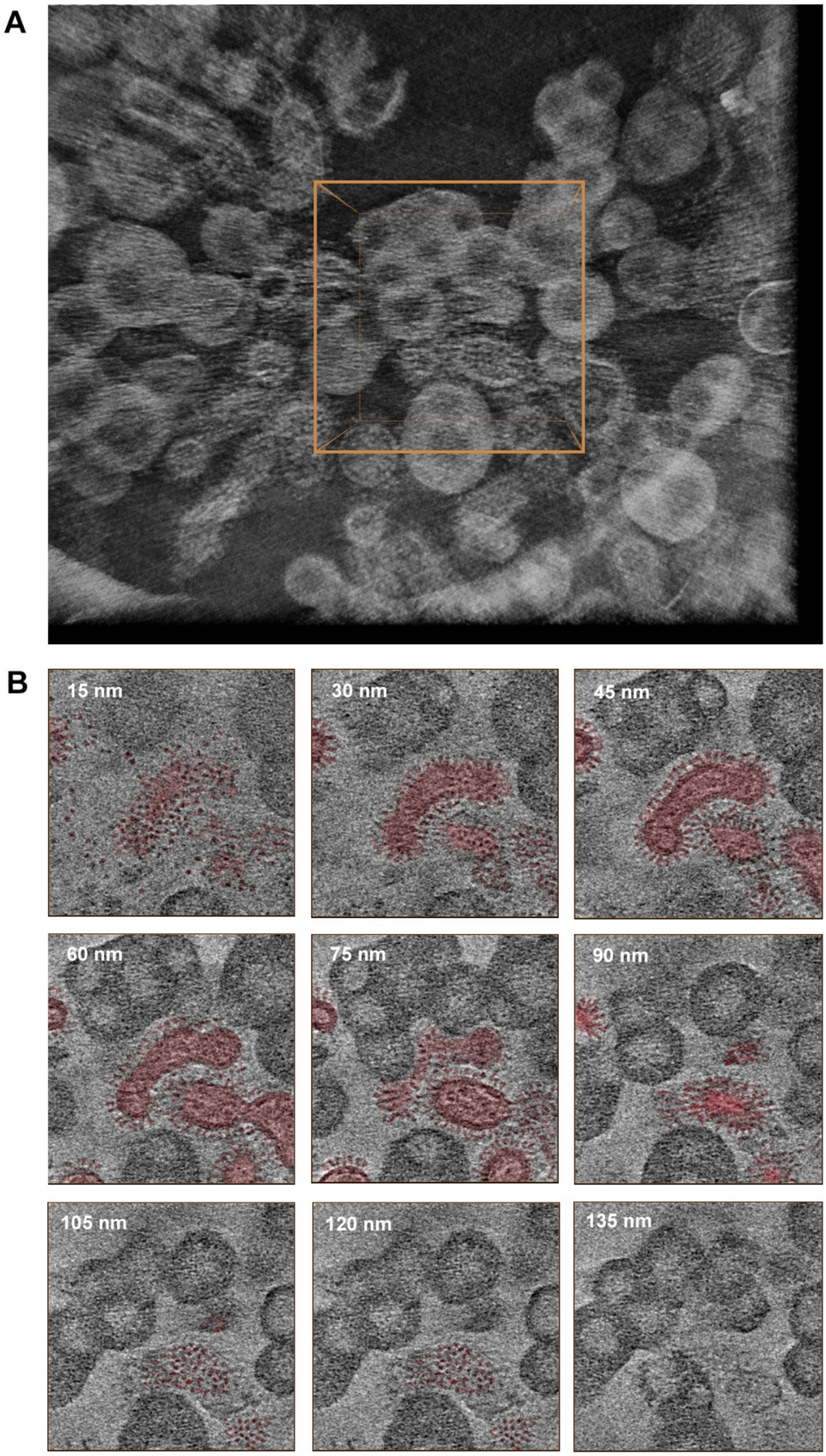

Figure S20. Cryo-electron tomography of F-NG 3 incubated with IAV X31 in PBS pH 7.4 for 30 min at room temperature: A) Reconstructed 3D volume of tomography tilt series (±65° at 2° angular increment) as a "voltex view" in ThermoFisher Amira™ software, the sub-volume, whose slices are shown in detail in B), is marked by a box. B) Cross sections (each 0.75 nm thick) through the sub-volume marked with a box in A, the distance between the shown slices is 15 nm. The virus particles are colored red for better recognition.

2.4. Cell-toxicity assay with nanogels

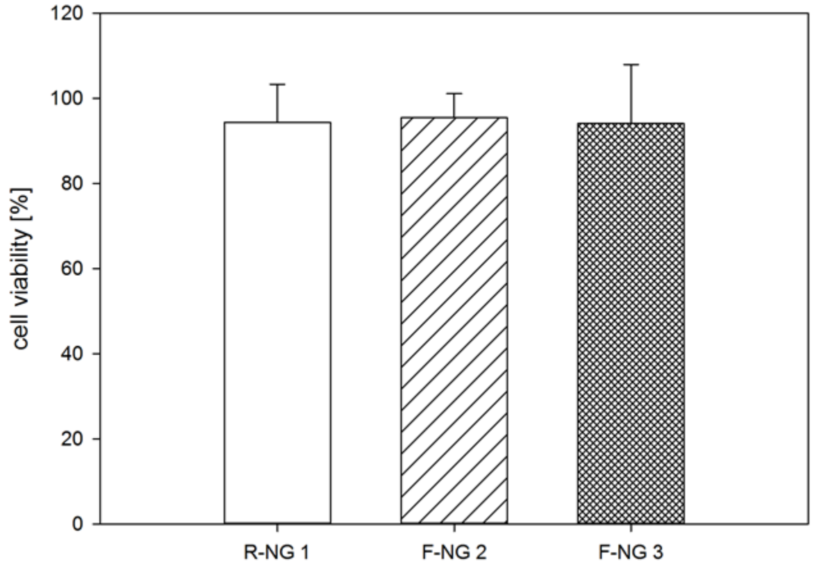

Figure S21. Cell viability of MDCK-II cells treated with 250 µg/ml NGs after 2 days of incubation at 37°C. Data are normalized to untreated cells (n=3). Error bars represent standard error of the mean.

2.5. AFM-PeakForce microscopy

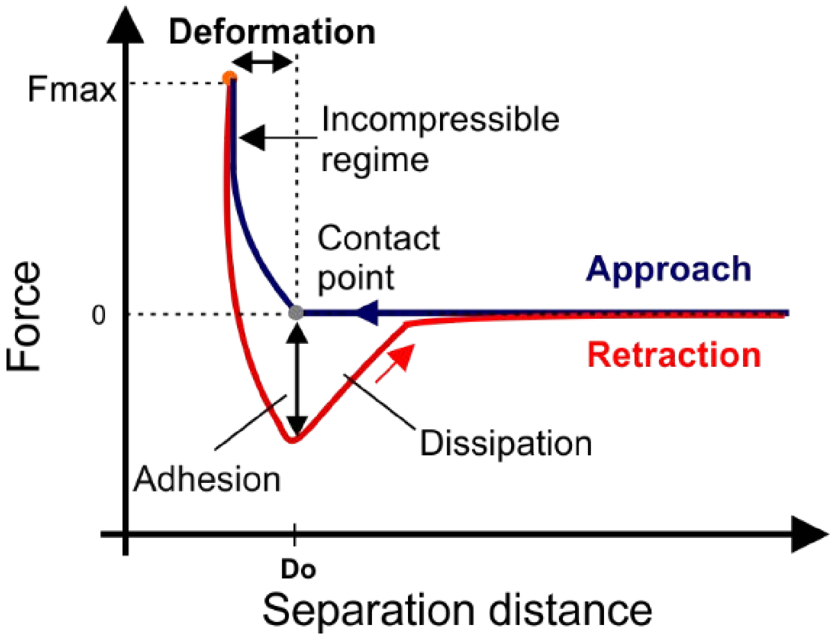

Figure S22. Schematic representation of the working principle of PeakForce imaging. Mechanical nanomapping is obtained on the fly through the application of repetitive approach-retraction cycles along the scanning direction. The plot shows the distinctive features present in a typical force-separation cycle between the tip and the sample.

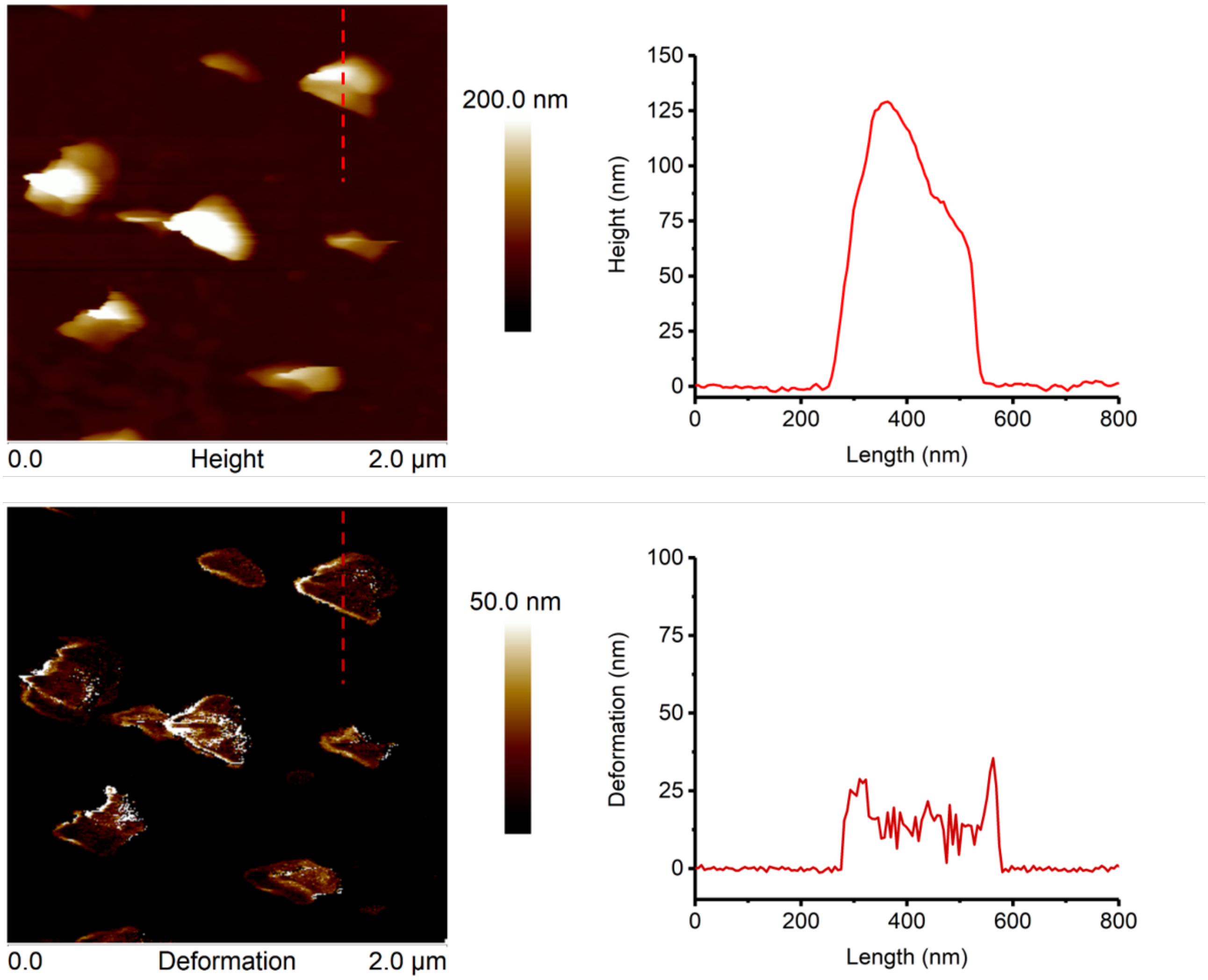

Figure S23. Surface topography and deformation maps of R-NGs1. Height images in a) show the morphology presented by F-NGs1 and a cross section profile of height is provided in b). In c) a color-coded map for the deformation attained by the AFM tip while imaging at a constant force is provided. In d) a cross section profile of deformation for a particular NG is given.

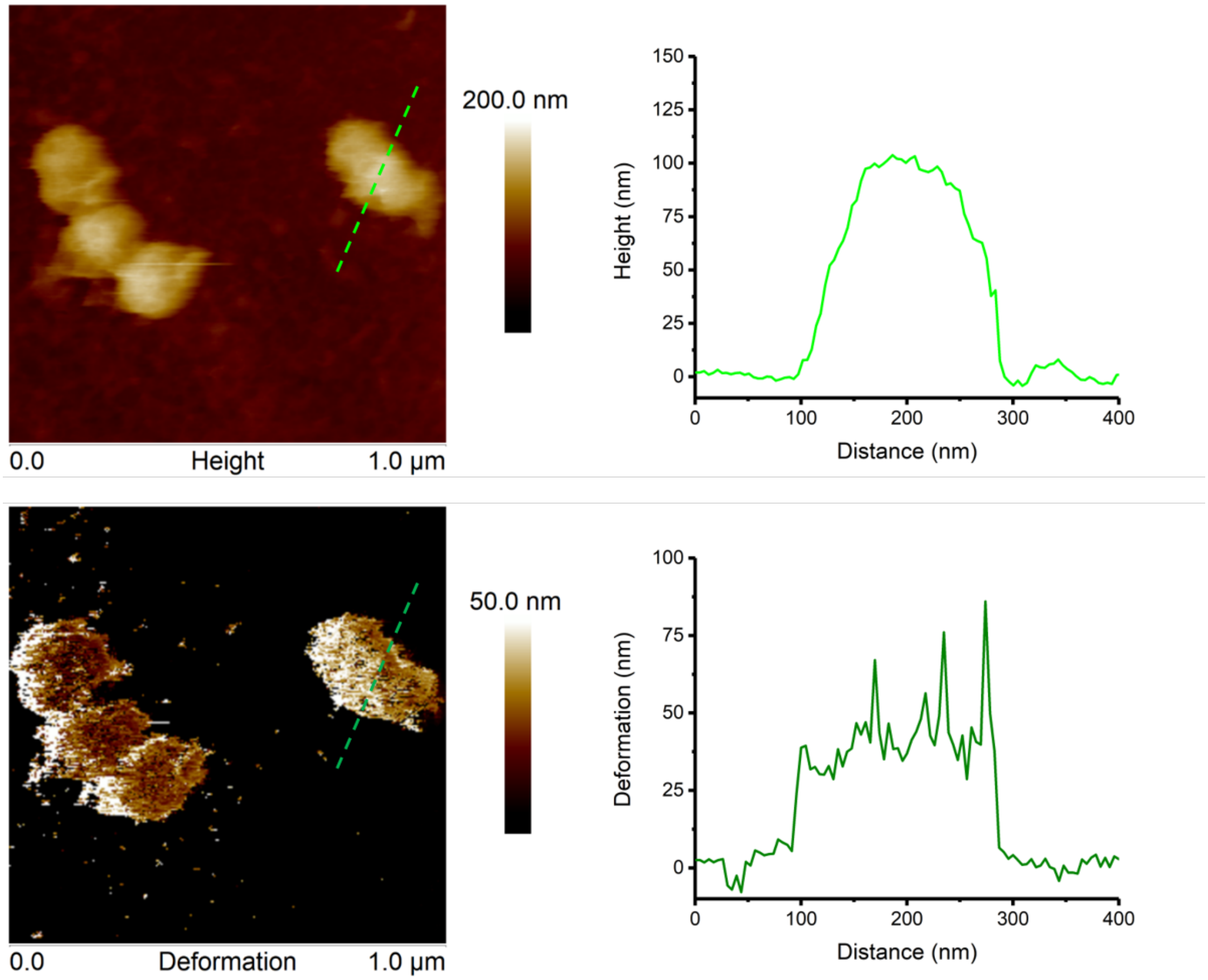

Figure S24. Surface topography and deformation maps of F-NGs2. Height images in a) show the morphology presented by F-NGs2 and a cross section profile of height is provided in b). In c) a color-coded map for the deformation attained by the AFM tip while imaging at a constant force is provided. In d) a cross section profile of deformation for a particular NG is given.

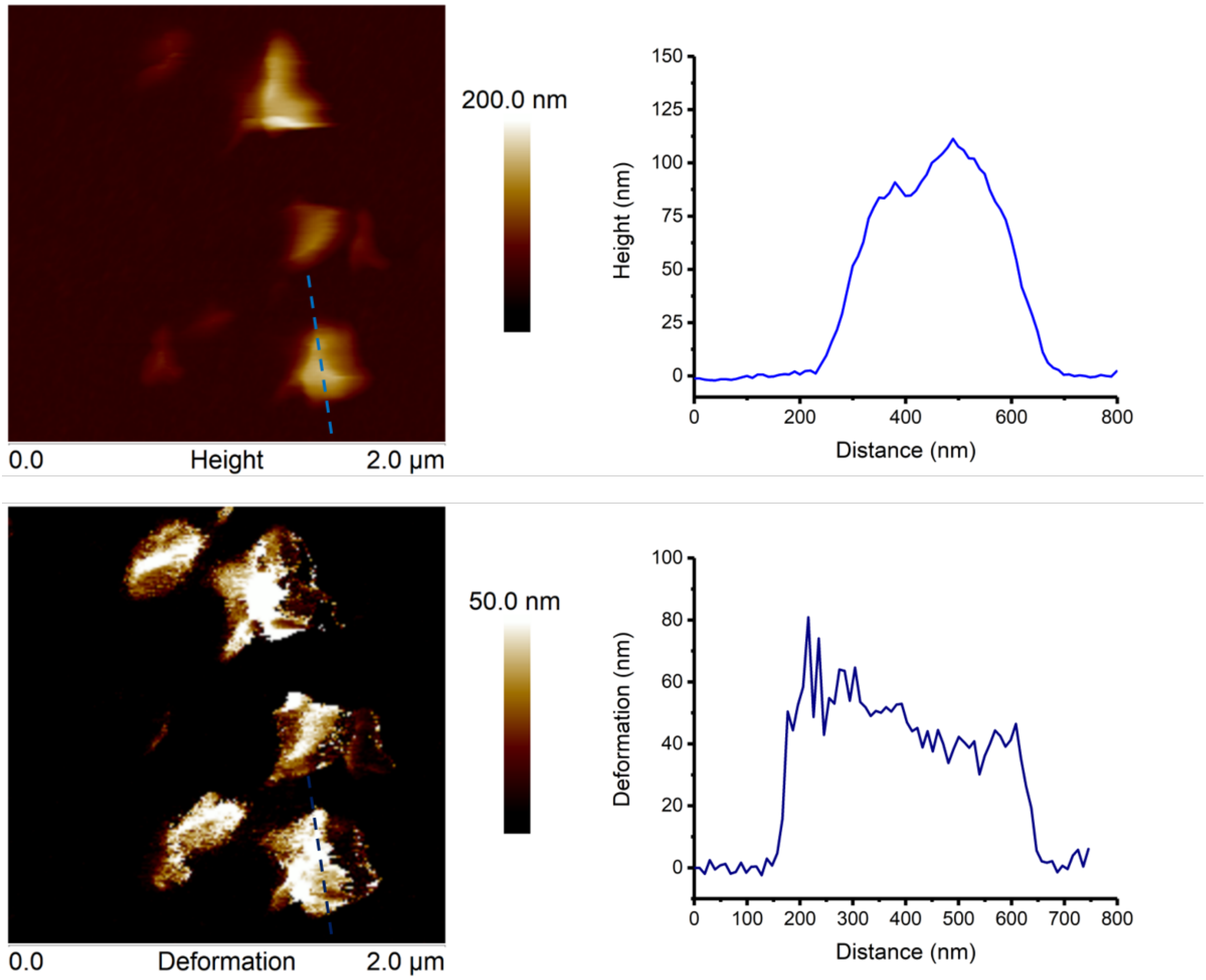

Figure S25. Surface topography and deformation maps of F-NGs3. Height images in a) show the morphology presented by F-NGs3 and a cross section profile of height is provided in b). In c) a color-coded map for the deformation attained by the AFM tip while imaging at a constant force is provided. In d) a cross section profile of deformation for a particular NG is given.

Point Nanoindentations.

Nanoindentations on individual NGs were performed after positioning the AFM tip directly on top of a previously imaged NG and by extending the z-piezo until a force of 20 nN was attained. In this way, force-distance curves are obtained and transformed into force-separation curves by subtracting the deflection of the cantilever to the piezo movement. In the model of contact of Sneddon, a semispace with a Young´s modulus E, is compressed by a conical indenter and the relationship between the applied Force F and the induced deformation δ, is given by

$$F = \frac{2E\tan(\alpha)}{\pi(1-\nu^2)}\delta^2$$

Where α is the half opening angle of the conical indenter and ν is the Poisson ratio for the NG material which accounts for the extent of expansion or shrinking in the direction perpendicular to the applied force. For the present case we take a value of $\alpha = 20^o$ for the half-opening angle of a SNL type tip, and a Poisson ratio of $\nu = 0.5$. Nanoindentations were taken with a tip velocity of 500 nm/s (approach and retraction) ad immediately retracted after reaching the set point.

2.6. Data for confocal image analysis

virion density (particles/mm^2)	control	C-NG	F-NG 3
	11318.77	3841.88	88.65876
	16904.27	4019.197	390.0986
	5821.925	5496.843	206.8704
Average	3103.057	3989.644	0
STD	9287.006	4336.891	171.4069

Inhibtion (%)	C-NG	F-NG 3
	58.63166	99.04535
	56.72236	95.79952
	40.81146	97.77247
	57.04058	100
Average	53.30152	98.15434
STD	8.368486	1.815795

2.7. NTA plots

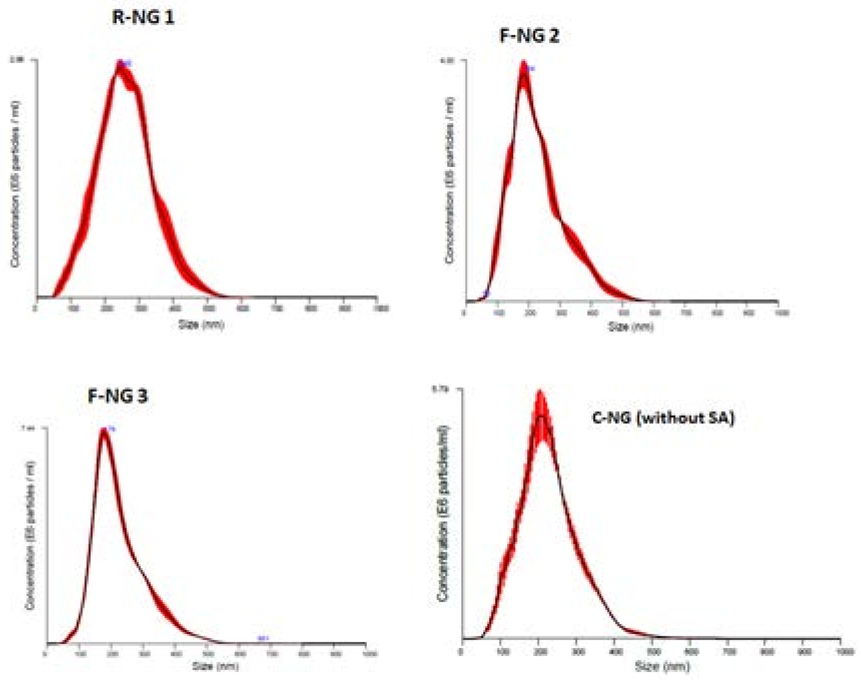

Figure S26. Size distribution profiles of all nanogels by NTA.

Table S1. Size and particle concentration analysis as observed in NTA.

Nanogel	Size by NTA (Mean ± SD)	Particle/mL for 1μg/mL concentration
R-NG 1	260 ± 82	5.7 E8
F-NG 2	226 ± 86	7.8 E8
F-NG 3	227± 81	1.1 E9
C-NG (without SA)	226 ± 75	4.3 E8

References

[1] M. Gervais, A. -L. Brocas, G. Cendejas, A. Deffieux, S. Carlotti, *Macromolecules* **2010**, *43*, 1778-1784.

[2] A. Sunder, H. Frey, R. Muelhaupt, *Macromol. Symp.* **2000**, *153*, 187-196.

[3] S. Bhatia, D. Lauster, M. Bardua, K. Ludwig, S. Angioletti-Uberti, N. Popp, U. Hoffmann, F. Paulus, M. Budt, M. Stadtmuller, T. Wolff, A. Hamann, C. Bottcher, A. Herrmann, R. Haag, *Biomaterials* **2017**, *138*, 22-34.

[4] S. Roller, H. Zhou, R. Haag, *Mol. Divers.* **2005**, *9*, 305-316.

[5] Z. Gan, R. Roy, *Can. J. Chem.* **2002**, *80*, 908-916.

[6] H. Ogura, K. Furuhata, M. Itoh, Y. Shitori, Carbohydr. Res. **1986**, *158*, 37-51.

[7] M. Müller, D. Lauster, H. H. K. Wildenauer, A. Herrmann, S. Block, Nano Lett. **2019**, *19*, 1875-1882.

[8] M. Bally, A. Gunnarsson, L. Svensson, G. Larson, V. P. Zhdanov, F. Höök, Phys. Rev. Lett. **2011**, *107*, 188103.

[9] D. W. Lee, H. L. Hsu, K. B. Bacon, S. Daniel, PloS one **2016**, 11(10), e0163437.

[10] E. T. Castellana, P. S. Cremer, Surface Science Reports **2006**, *61*, 429 - 444.

[11] U. Desselberger, *Arch. Virol*. **1975**, *49*, 365-372.
